# Endosidin 2 accelerates PIN2 endocytosis and disturbs intracellular trafficking of PIN2, PIN3, and PIN4 but not of SYT1

**DOI:** 10.1371/journal.pone.0237448

**Published:** 2020-08-13

**Authors:** Alexandra Lešková, Mária Labajová, Miroslav Krausko, Alexandra Zahradníková, František Baluška, Karol Mičieta, Ján Turňa, Ján Jásik

**Affiliations:** 1 Institute of Botany, Plant Science and Biodiversity Center, Slovak Academy of Sciences, Bratislava, Slovakia; 2 Institute of Experimental Endocrinology, Biomedical Research Center, Slovak Academy of Sciences, Bratislava, Slovakia; 3 Institute of Cellular and Molecular Botany, University of Bonn, Bonn, Germany; 4 Department of Botany, Faculty of Natural Sciences, Comenius University, Bratislava, Slovakia; 5 Comenius University Science Park, Comenius University, Bratislava, Slovakia; 6 Department of Molecular Biology, Faculty of Natural Sciences, Comenius University, Bratislava, Slovakia; Iowa State University, UNITED STATES

## Abstract

We established that Endosidin2 (ES2) affected the trafficking routes of both newly synthesized and endocytic pools of PIN-FORMED2 (PIN2) in Arabidopsis root epidermal cells. PIN2 populations accumulated in separated patches, which gradually merged into large and compact ES2 aggregates (ES2As). FM4-64 endocytic tracer labeled ES2As as well. Both PIN2 pools also appeared in vacuoles. Accelerated endocytosis of PIN2, its aggregation in the cytoplasm, and redirection of PIN2 flows to vacuoles led to a substantial reduction of the abundance of this protein in the plasma membrane. Whereas PIN-FORMED3 and PIN-FORMED4 also aggregated in the cytoplasm, SYT1 was not sensitive to ES2 treatment and did not appear either in the cytoplasmic aggregates or vacuoles. Ultrastructural analysis revealed that ES2 affects the Golgi apparatus so that stacks acquired cup-shape and even circular shape surrounded by several vesicles. Abnormally shaped Golgi stacks, stack remnants, multi-lamellar structures, separated Golgi cisterna rings, tubular structures, and vesicles formed discrete clusters.

## Introduction

Directional flow of membrane material between sub-cellular compartments by different vesicular structures is essential for fundamental cellular functions such as cell division, polarization, differentiation, motility, and response to environmental stress. A pharmacological approach using chemical compounds combined with the monitoring of membrane proteins labeled by fluorescence proteins or visualized by immunohistology techniques has become a favorite method to study dynamics in plant cell membrane structures. Many new bioactive small molecules with an impact on the vesicle trafficking network have been discovered by high-throughput chemical genetic screens [1–5]. However, mostly precise targets for these bioactive compounds have not been identified. One of the exceptions is Endosidin2 (ES2; 3-Fluorobenzoic acid [(4-hydroxy-3-iodo-5-methoxyphenyl)methylene]hydrazide) and its more active analog ES2-14 that have been shown to interfere with the Arabidopsis EXO70A1 protein in DARTS, MST, DSF, and NMR chemical shift assays [6,7]. EXO70 members are subunits of the exocyst, an octameric protein complex involved in the secretion most likely by targeting post-Golgi vesicles [8,9]. In Arabidopsis, the influence of ES2 resulted in the cytoplasmic aggregation of proteins such as PIN-FORMED1, PIN-FORMED2 (PIN2), and BRASSINOSTEROID-INSENSITIVE1 [6]. It was concluded that ES2 has the potential to be widely used in membrane trafficking studies and clinical practice, including cancer research [6,7]. Notably, ES2-14 was also demonstrated to be an efficient agent in suppressing fungal growth and virulence [7]. To better understand the effects of ES2, we examine the influence of this new attractive compound on the trafficking of PIN2 and several other Arabidopsis plasma membrane (PM) proteins using a photoconvertible fluorescence protein technique. This approach, which enables distinguishing between the effects of compounds on the secretory and endocytic trafficking routes of PM proteins simultaneously, has proved to be very useful in the investigation of the PIN2 auxin efflux carrier in our previous studies [10–12]. We wished further to develop this system and study if ES2 influences both PIN2 trafficking routes. Finally, we wanted to examine the effects of ES2 on the ultrastructural level in epidermal root cells.

## Results

### ES2 influences PIN2 trafficking

**Fig 1:**
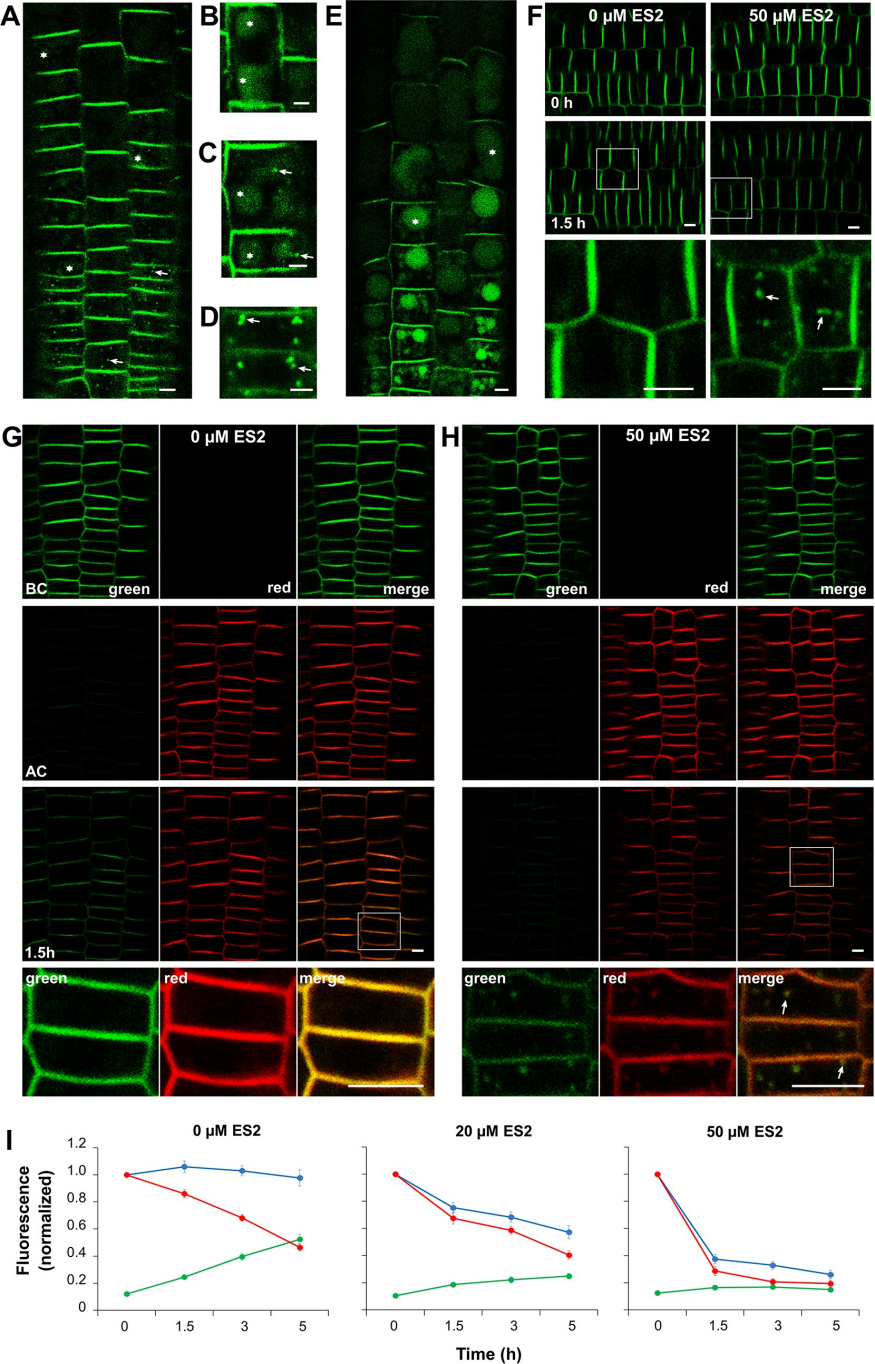
Endosidin2 influences the dynamics of newly synthesized and endocytosed PIN2 populations. The root treated with 50 μM ES2 for one hour in the experiment without photoconversion is shown in A. A fluorescence signal is evident in cytoplasmic ES2As (arrows) and vacuoles (asterisks). Clear ES2As are present in meristematic cells (arrows in D) and cells of the root transition zone (arrows in C). Vacuoles are labeled in the cells of the transition zone (asterisks in C) and the elongation zone (asterisks in B). After three hours of ES2 treatment, vacuoles are strongly fluorescent over the entire root (asterisks in E). The experiment without photoconversion shows that ES2 reduces the abundance of PIN2 protein in the PM (F and I, arrows in F point to ES2As). The photoconversion experiment indicates that ES2 accelerates disappearing the old red PIN2 population from the PM and inhibits the recovery of the newly synthesized green PIN2 pool in the PM (G-I). In this experiment, roots were imaged in green and red channels (BC), converted, then again pictured (AC) and transferred onto medium without (G) or with 50 μM ES2 (H) and reimaged at different time points. G and H show representative images captured during the time-lapse experiment. The detailed image, shown at high magnification and detector sensitivity, confirms the appearance of both red internalized and green newly synthesized PIN2-Dendra2 pools in ES2As (arrows). Charts (I) show changes in signal intensity of the PM in a time-lapse experiment without photoconversion (blue lines) and changes in red (red lines) and green fluorescence intensities (green lines) of the PM in the photoconversion experiment. The means of the PM's green signal intensities at each time point in the trial were normalized to the mean of PM green signal intensities of the same root assessed before conversion. Time-points 0 represent the relative green signal intensities recorded immediately after conversion. The means of red signal intensities at each time point during the experiment were related to the mean red fluorescence intensity measured immediately after conversion (time-point 0). In parallel non-photoconversion experiments, the means of green signal intensities at each time point in the trial were normalized to the mean of green signal intensity of the PM of the same root before application of treatment. Values of the time-points 0 were set to 1. The two-way ANOVA test showed that both, effects of ES2 concentrations and duration of treatment were statistically highly significant (p ≤ 0.001, n = 15) in all events (green signal intensity in the experiment without photoconversion and green and red signal intensities in the photoconverting experiment). Bars = 5 μm.

At first, we wished to confirm using the PIN2-Dendra2 transgenic line [10] two previous findings obtained with the usually employed PIN2-GFP construct, i.e., that PIN2 appears in cytoplasmic aggregates and within vacuoles. Indeed, after seedling treatment with 50 μM ES2, we observed in a routine time-laps experiment without photoconversion the development of discrete ES2 agglomerates (ES2As) in the cytoplasm (Fig 1A) with about 1 μm size. The PIN2-Dendra2 fusion protein also appeared in large, mostly round structures resembling vacuoles (Fig 1A) even when seedlings were permanently kept in the light. Although ES2As were visible in the meristem zone, vacuolar labeling was not apparent in these cells (Fig 1A–1D). Cells in the root apex transition zone, as characterized previously [13], exhibited the fluorescence in both, vacuoles and ES2As (Fig 1A–1C). The PIN2-Dendra2 signal was weaker in the vacuoles than in ES2As (Fig 1C, S1A and S1C Fig). Labeling within the vacuoles was obvious in root elongating cells (Fig 1B), which became more prominent with extended treatment (Fig 1E). Analysis of PIN2-Dendra2 dynamics in the plasma membrane (PM) by time-lapse experiment highlighted decreasing signal intensity compared to the controls exhibiting the constant fluorescence (Fig 1F–1I). We then performed photoconversion experiments to determine which of the PIN2-Dendra2 routes is affected by the ES2 treatment, internalization of the protein from the PM, or externalization of the newly synthesized protein. The roots were illuminated by the Hg arc lamp as we previously described [10], and the dynamics of both red and green PM signals were estimated in a time-lapse trial. We discovered a substantial decline in red signal intensity and failure of green signal recovery in the PM after ES2 treatment (Fig 1G–1I), and these effects were ES2 concentration-dependent (Fig 1I). Further, we have found that both red internalized and green newly synthesized PIN2-Dendra2 pools are gathered in ES2As (Fig 1H).

### Newly synthesized and PM derived PIN2 populations appear in vacuoles following ES2 treatment

**Fig 2:**
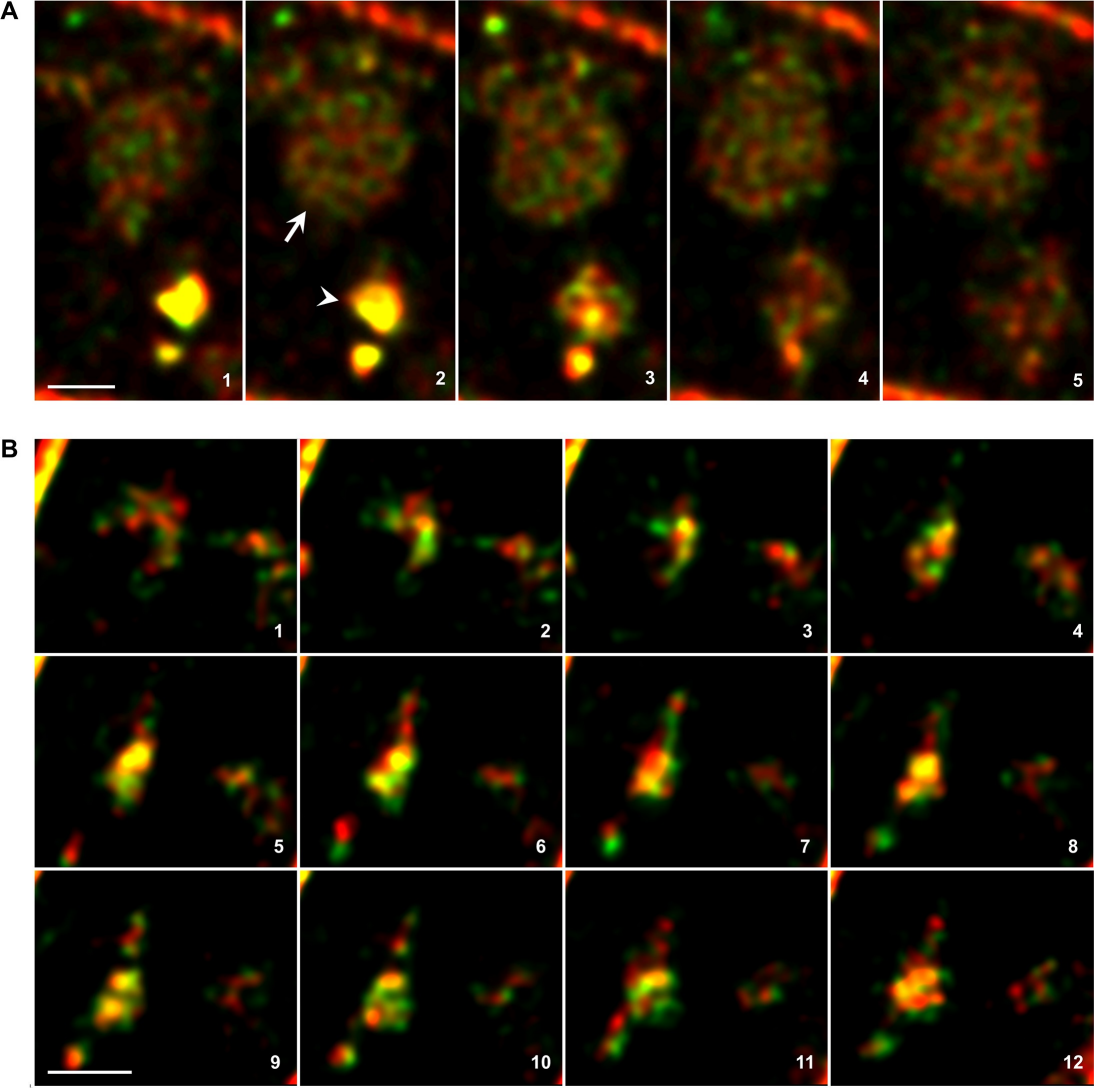
Newly synthesized and internalized PIN2 populations appear in ES2As and vacuoles. In the photoconversion experiment, vacuoles (arrow) and ES2As (arrowhead in A) are detectable in both green and red channels. Note that the green and red signals were partly overlain in large ES2As (yellow color). B shows detailed images of the cytoplasm area with green and red separated patches. These merge into larger aggregates where the signals partly overlap (yellow color). In this experiment, roots were photoconverted, then seedlings were placed on the medium with 50 μM ES2 for 1.5 hours and imaged with a Leica TCS SP8 STED 3X microscope. The numbers in the merged images combining red and green channels indicate optical slices' order in the Z-stack. Bars = 1 μm.

To confirm that the large round structures observed in the green channel after the ES2 treatment are indeed vacuoles, we treated seedlings with 2 μM styryl membrane dye FM 4–64 (N-(3-Triethylammoniumpropyl)-4-(6-(4-(Diethylamino) Phenyl) Hexatrienyl Pyridinium Dibromide) and 50 μM ES2. Shortly after treatment, the FM 4–64 vital fluorescence probe should be present in the PM. Shortly after treatment, this vital fluorescence probe should be present in the PM; after internalization, it should label endocytic intermediates between the PM and the vacuole, and later on, the dye should stain the tonoplast and enter vacuoles [14]. As expected, we detected round sectors containing PIN-Dendra2 fusion in the green channel following ES2 application. These compartments were surrounded by an envelope revealed in the red channel (S1C Fig). We did not observe the green signal in vacuoles either in wild-type plants after application of ES2 or in the light growing PIN2-Dendra2 seedlings without ES2 treatment. Then we analyzed vacuole fluorescence in photoconversion mode by advanced Leica TCS SP8 STED 3X microscope to determine which PIN2 population moved to the vacuoles. After photoconversion and treatment of the roots with 50 μM ES2 for 1.5 hours, we detected both green and red fluorescence signals in the vacuoles, and these did not entirely overlap (Fig 2A).

### PIN2 populations form diverse patches that merge in large ES2As

**Fig 3:**
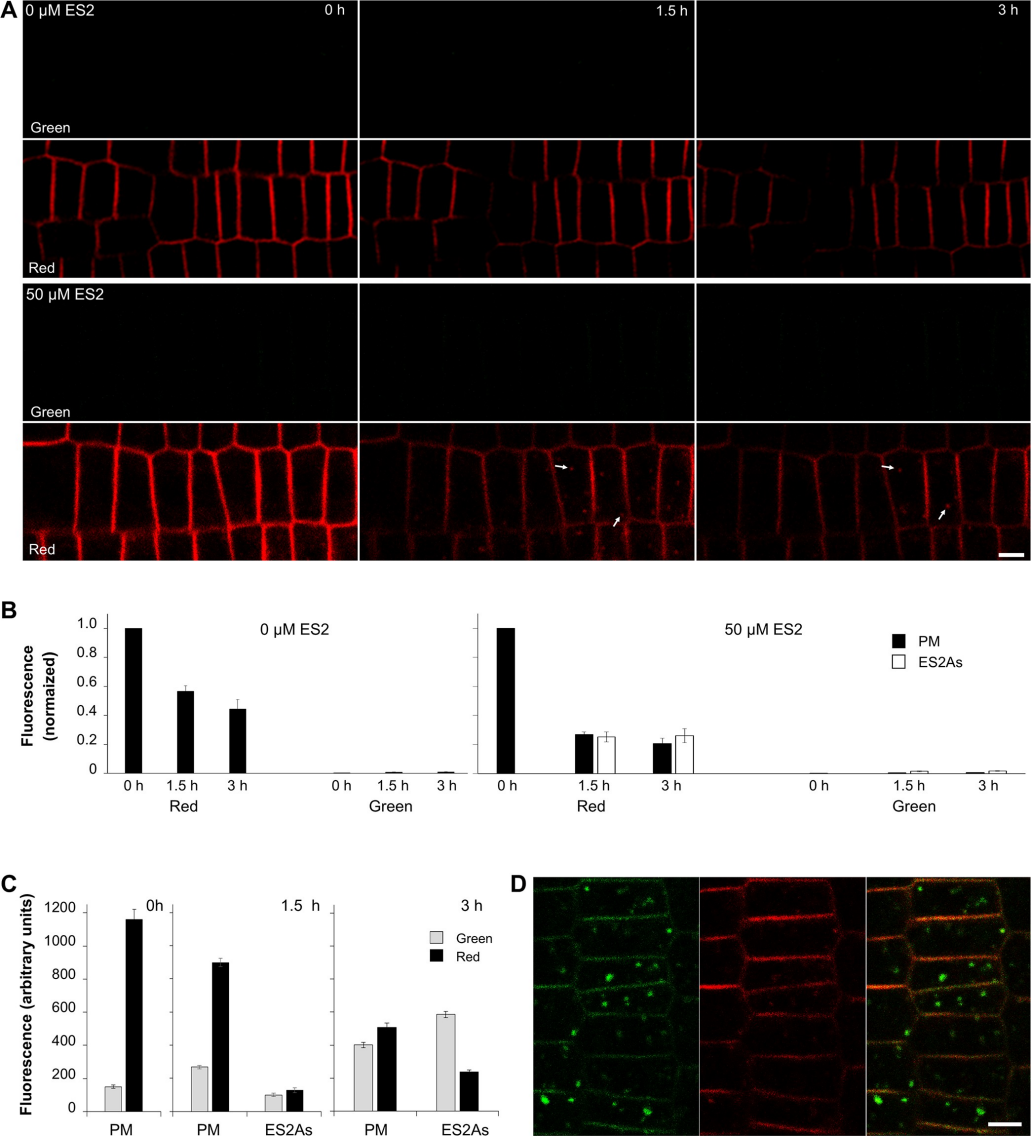
The red PIN2 population in ES2As is of the PM origin. In the presence of 50 μM cycloheximide, intra-cytoplasmic aggregates contain only the red PM-derived PIN2 population (A). The B charts show the normalized fluorescence intensities of the PM and ES2As. In this experiment, seedlings were pre-treated with cycloheximide for 30 minutes, then photoconverted and placed on the medium with 50 μM cycloheximide and with or without 50 μM ES2. Roots were immediately imaged and then reimaged after 90 minutes and three hours. The means of the green signal intensities of the PM and ES2As at each time point were normalized to the mean of green signal intensities of the PM of the same root before photoconversion. Time-points 0 represent the relative green signal intensities recorded immediately after conversion. The means of red signal intensities at each time point during the experiment were related to the mean of red fluorescence intensities of the PM measured immediately after conversion (time-point 0). Values of the time-points 0 were set to 1. Note that ES2As are only detectable in the green channel. In the experiment shown in C and D, seedlings were photoconverted and kept on the standard cultivation medium for two hours and then placed on the medium with ES2, imaged and then reimaged after 1.5 and three hours. Note ES2A accumulation of both red and green PIN2 populations. The charts in C show fluorescence intensities of ES2As and the PM in green and red channels. D depicts the representative image of cells in the meristematic zone of the root taken after three hours of ES2 treatment. Bars = 5 μm.

ES2 should interfere with the EXO70 subunit of the exocyst [6,7]. This complex is involved in the exocytic pathway by targeting post-Golgi vesicles to the PM [8,9]. Therefore, we expected that the newly synthesized PIN2 population devoted to secretion would accumulate in the ES2As. We confirmed this, but surprisingly also the red signal appeared in these intra-cytoplasmic inclusions (Fig 1H, Fig 2, S2 Fig). Noteworthy, whole root tips are photoconverted in routine photoconversion experiments by epifluorescence microscopy, and one cannot exclude that the red form of PIN2-Dendra2 is the newly synthesized protein population and not of the PM origin. The mature green PIN2-Dendra2 variety, if still present in the endomembrane system, also converts to the red form after photoconversion. This population can subsequently aggregate in ES2As with the green protein population synthesized after photoconversion. To confirm that the red population has PM origin, we pre-treated seedlings with 50 μM cycloheximide (CHX) for 30 minutes, illuminated roots for photoconversion, and treated seedlings with 50 μM ES2 in the presence of CHX. We used a similar procedure in our previous study with BFA [12], and the approach was recently employed in the study with the classic PIN2-GFP construct [15]. Herein, detected ES2As labeled by the red PIN2 variety, but the green PIN2-Dendra2 population was absent in these aggregates and the PM (Fig 3A and 3B). In the alternative experiment, we photoconverted the roots and kept them on the standard cultivation medium. After two hours, the part of the red PIN2 population in the PM was already replaced by the newly synthesized green variety (Fig 3C, see also our previous studies [10–12]). After such an extended period after photoconversion, all remnants of the red newly synthesized PIN2-Dendra2 variety potentially present in the endomembrane system should already be translocated to the PM. We consequently treated these seedlings with 50 μM ES2 and observed both red and green PIN-Dendra2 varieties in ES2As (Fig 3C and 3D). These experiments confirmed that the red PIN2-Dendra2 population in ES2As has the PM origin and that ES2 also affects the intracellular route of internalized PIN2.

To characterize in detail the development of ES2As, we analyzed cells in the Z-stack modus at higher magnification and sensitivity by advanced Leica TCS SP8 STED 3X microscopy. In the experiment without photoconversion after Huygens deconvolution, we detected small loosely packed and weakly-fluorescent patches present together with the compact and strongly fluorescent ES2As previously visible under the Olympus confocal laser microscope in the routine time-lapse experiments. Next, we used the photoconversion approach. We identified spots either in green or red channels, but there were also clumps with partly overlapping signals located nearby (Fig 2B, S2 Fig). Further, we found that in large ES2As, both the green and red signals were much more intensive and largely overlain (Fig 2A, S2 Fig). ES2As were randomly distributed in the cytoplasm and occasionally visible in the vacuoles, or their close vicinity (S2 Fig).

### PIN2 pools assemble with the FM4-64 endocytic tracer in ES2As

**Fig 4:**
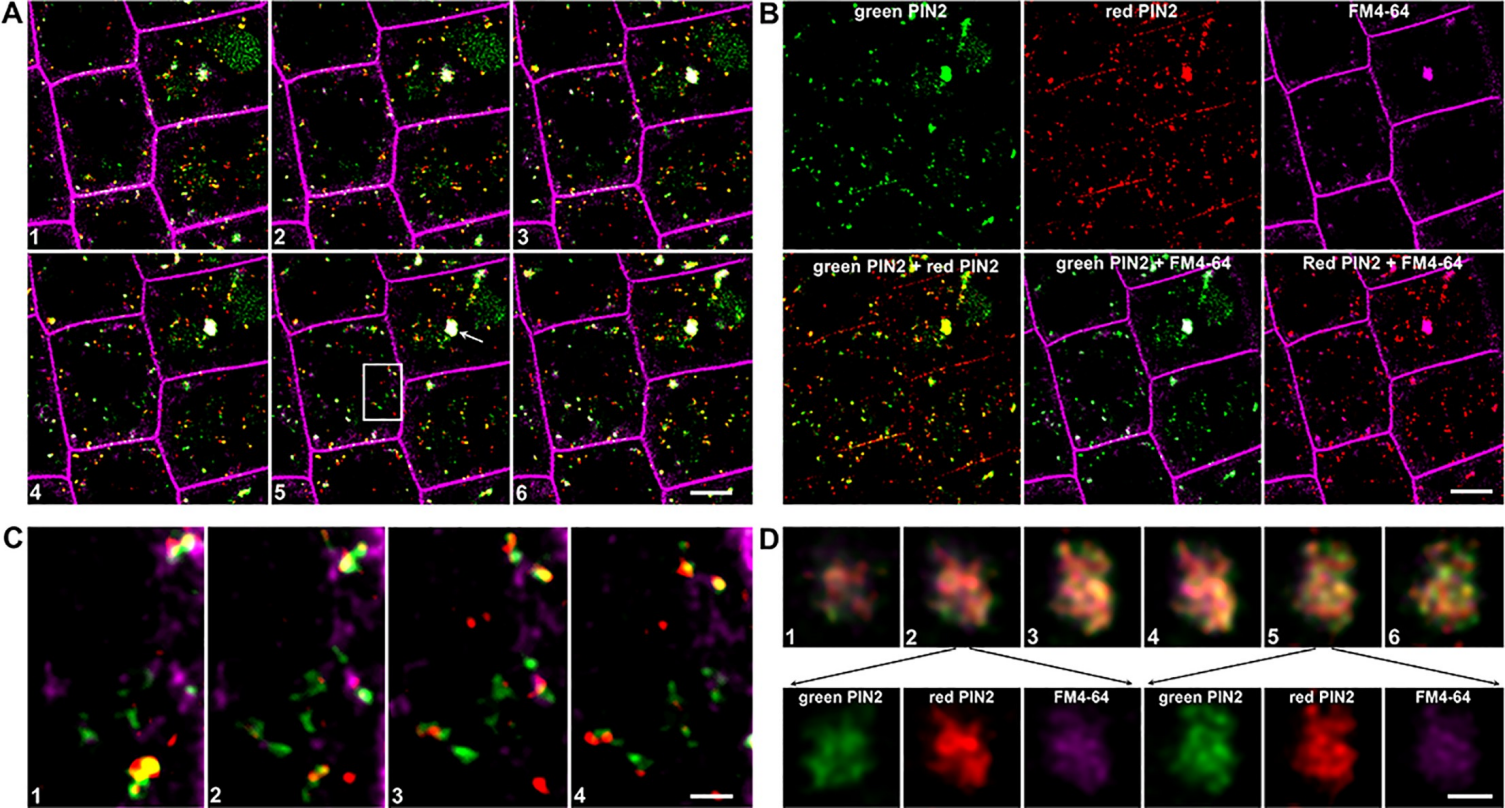
PIN2 populations assemble with FM4-64 endocytic tracer in large ES2As but not in smaller bodies. After photoconversion and co-treatment of seedlings with 50 μM ES2 and 2 μM FM4-64 for 1.5 hours, small differently fluorescent patches and large aggregates were detectable in the cytoplasm (A). B shows separate and merged channels of optical slice No. 5; the outlined area is shown at higher magnification in C, the large ES2A marked with an arrow is displayed in D at higher magnification and in separate and merged channels. Note that the aggregate is visible in all three fluorescence channels; nevertheless, signals did not entirely overlay. The numbers in the images indicate the order of optical slices in the Z-stack. Bars = 5 μm in A and B, = 1 μm in C and D.

Then we applied the FM4-64 endocytic tracer. In the experiment without photoconversion, small green and red fluorescence patches appeared in the cytoplasm after co-treating the cells with 50 μM ES2 and 2 μM FM4-64 for 30 minutes, and these patches occasionally overlapped (S1B Fig). We also observed relatively large and compact ES2As in both red and green channels after extended treatment (S1C Fig, S3 Fig). We then performed the test in photoconversion mode. We set the Leica TCS SP8 STED 3X microscope so that imaging could distinguish the signals emitted by FM4-64 and PIN2-Dendra2 populations. This approach detected small spots in all three channels, and signals were occasionally overlapped (Fig 4A–4C). We then analyzed the degree of signal colocalization in cytoplasm areas enriched in fluorescent patches, and this established that the calculated Pearson correlation coefficients quantifying the overlap between individual fluorophores were relatively low (r_green PIN2 vs. FM4-64_ = 0.37±0.16; r_red PIN2 vs. FM4-64_ = 0.31±0.10; r_green PIN2 vs. red PIN2_ = 0.60±0.03). Large, compact, and strongly fluorescent ES2As are detectable in all three channels (Fig 4A–4D). The green and both red signals in these structures were not wholly overlain (Fig 4D), but the degree of signal colocalization was quite high and similar (r_green PIN2 vs. FM4-64_ = 0.85±0.02; r_red PIN2 vs. FM4-64_ = 0.80±0.02; r_green PIN2 vs. red PIN2_ = 0.83±0.03 As the hypothesis that the endocytic PIN2-Dendra2 population would entirely overlap internalized FM4-64 tracer, had proved wrong, we performed additional tests. We co-treated seedlings with 50 μM ES2 and 2 μM FM4-64 in the presence of 50 μM cycloheximide to block protein synthesis, and this also determined that the small green and red bodies visible 30 minutes after ES2 treatment were separate entities (S4A Fig). Although the large compartments visible after more extended ES2 treatment show both red and green signals, these also did not completely overlap (S4B Fig, r = 0.80±0.03). These experiments indicate that FM4-64 and PIN2 internalize to different pools of early endosomes, and these are gathered in ES2As after ES2 treatment.

### The effects of ES2 are partly reversible

To determine if ES2 suppression of membrane protein trafficking is a reversible process and its impact it has on seedling development, we treated seedlings with 50 μM ES2 for two hours. We observed a substantial decrease in the PM green signal intensity and the appearance of numerous ES2 bodies in the cells (S5A and S5B Fig). When seedlings were then rinsed with the medium without ES2 and then kept on ES2 free medium, the PIN2 containing aggregates disappeared from the cells within an hour (S5A–S5C Fig), and there was a subsequent increase in the PM green signal intensity (S5A, S5C and S5D Fig). However, the PIN2 protein steady-state remained at a lower level than the protein abundance recorded at the start of the experiment. ES2s overall destructiveness and inability of the seedlings to revitalize after this treatment are documented in the following root growth experiment. We treated 4-day-old seedlings with 20 or 50 μM ES2 for two hours, and the entire cultivation period. We then analyzed root increments after 24 and 48 hours, and this highlighted significant root growth restriction after the short two-hour ES2 pulse and when seedlings were kept permanently on ES2 medium (S5E and S5F Fig).

### ES2 and BFA cause formation of distinct aggregates

Brefeldin A (BFA), a fungal lactone, causes similarly to ES2 agglomeration of both endocytic and exocytic PIN2 populations in the cytoplasm [12]. We wished to compare the developmental characteristics of compartments induced with these two compounds and study how reciprocal pretreatment and co-treatment affect the aggregation of PIN2. When seedlings were treated with BFA at the commonly used 50 μM concentration, the BFA compartments (BFACs) appeared 15–20 min after BFA application. S6A and S6C Fig show that although these BFACs were initially relatively small and abundant, they quickly enlarged, and there were then only 1–2 per cell (S6D Fig). The enlarged BFACs were obviously formed by clustering small bodies observed shortly after the BFA application. Moreover, BFACs size was not dependent on BFA concentration (S6C Fig; see also our previous study [12]). Transmission electron microscopy (TEM) identifies massive vesicular clusters in the cells (arrows S6D Fig). When we subjected seedlings to ES2 treatment and used the standard microscopy imaging for time-lapse experiments, ES2As were visible approximately 30 minutes after the ES2 application. They were abundant (8.66 ± 0.37 ES2As per cell on the optical section), relatively small, varied in size, and mostly irregularly shaped. ES2As remained tiny and abundant after extended treatment, regardless of the applied ES2 concentration (S6B and S6C Fig). In the additional experiment, we kept seedlings on the medium with ES2 for 1.5 h and subsequently on the medium with ES2 and BFA (both at 50 μM) and vice versa. When seedlings were treated with ES2 and then with ES2 and BFA, we failed to observe massive bodies, but small spots were visible (S6F Fig). In the reciprocal experiment after treatment seedlings with BFA and following co-treatment with BFA and ES2, we detected large fluorescent compartments and small spots (S6G Fig). Large compartments were frequently irregularly shaped.

### ES2 disrupts the Golgi/trans-Golgi organelles

**Fig 5:**
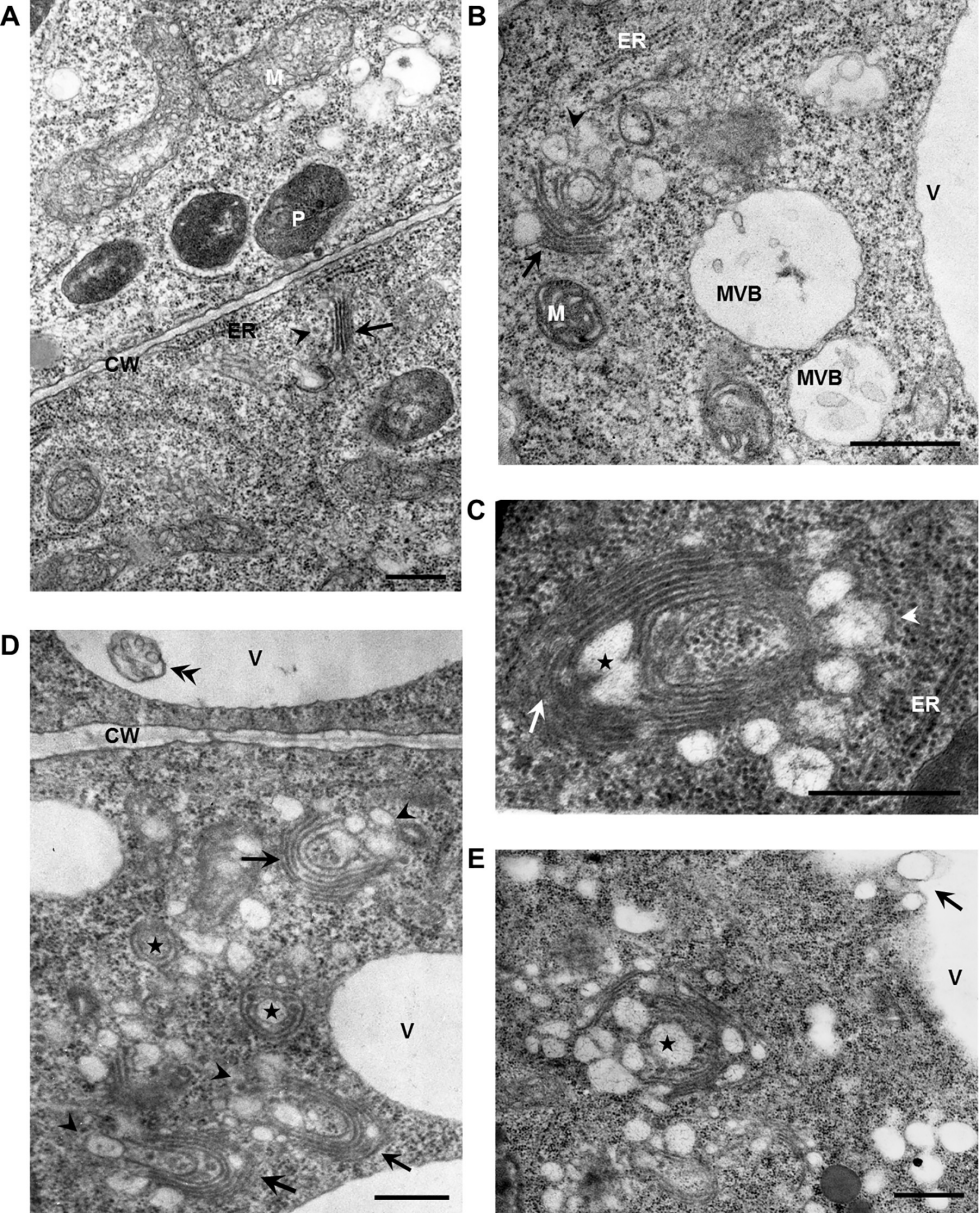
ES2 affects the Golgi apparatus. In control root epidermal cells, the Golgi apparatus consists of stacks of a few flattened, straight cisternae (A). ES2 causes accumulation of enlarged vesicles near the Golgi (B) and even in Golgi stacks (asterisk in C), Golgi stack expansion, bending, circulation (B-D), and separation of encircled Golgi cisternae (asterisks in D). Aberrant Golgi units congregate (D), and further degrade so that large compartments composed of a heterogeneous mixture of multi-lamellar structures and vesicles (asterisks in E) are visible in the cytoplasm (E). Arrows in A-D point to Golgi cis face and arrowheads to trans-face. Note the formation of multi-vesicular bodies in the vicinity of aberrant Golgi (B), their appearance in the vacuole (double arrowhead in D), and the release of vesicle complexes to vacuoles (arrow in E). M = mitochondria, MVB = multivesicular body, CW = cell wall, V = vacuole, ER = endoplasmic reticulum, P = plastid. Bars = 0.5 μm.

We inspected root epidermal cells by TEM to establish which organelles are affected by ES2 treatment and which structures are involved in the formation of ES2As. The typical Golgi complex in the control interphase epidermal cells consists of stacks of a few flattened, straight, tightly packed cisternae, vesicles with 50 nm diameter at the trans-Golgi region periphery and an indistinct trans-Golgi network (TGN, Fig 5A). In the experiment with 50 μM ES2, the accumulation of enlarged vesicles near the Golgi was evident after 30 minutes. After an hour of ES2 treatment, we observed 4.79 vesicles (± 1.42 SD, n = 45) with 84.33 (± 28.9 SD, n = 150) nm diameter. Vesicles were predominantly located near the trans-Golgi (Fig 5B), but they frequently surrounded the whole Golgi stack or were inside these stacks (Fig 5C and 5D). Vesicles contained fine precipitates in their lumens (Fig 5C–5E), and some vesicle clusters were developed as multi-vesicular bodies (Fig 5B). The Golgi cisternae were expanded, and whole stacks were enlarged and bent into cup-shapes or multi-lamellar circles with 0.414 μm diameter (± 0.088 SD, n = 45, Fig 5B, 5C and 5D). The Golgi stacks frequently disintegrated, cisternae separated, and consequently, distinct rings of Golgi cisternae were detected in close vicinity to stack remnants (Fig 5B–5D). Aberrant Golgi and their derivatives formed distinct spherical aggregates; two or more such units very frequently assembled into more complex forms (Fig 5D). After 2 hours of 50 μM ES2 treatment, the Golgi structures were difficult to recognize in conglomerates consisting of stack remnants and vesicles. Moreover, the vesicle complexes were often released into the vacuoles (Fig 5E). Other cell structures, such as endoplasmic reticulum, remain unaffected by ES2 (Fig 5B and 5C). In cells treated with 50 μM BFA, we observed typical 1–2 large BFACs (S6E Fig) consisting of an enormous number of vesicles (more than 200 within a section through the most massive BFACs) with a diameter of 91.64 nm (± 21.26 SD, n = 150).

### ES2 influences trafficking routes of other PINs, but not SYT1 protein

**Fig 6:**
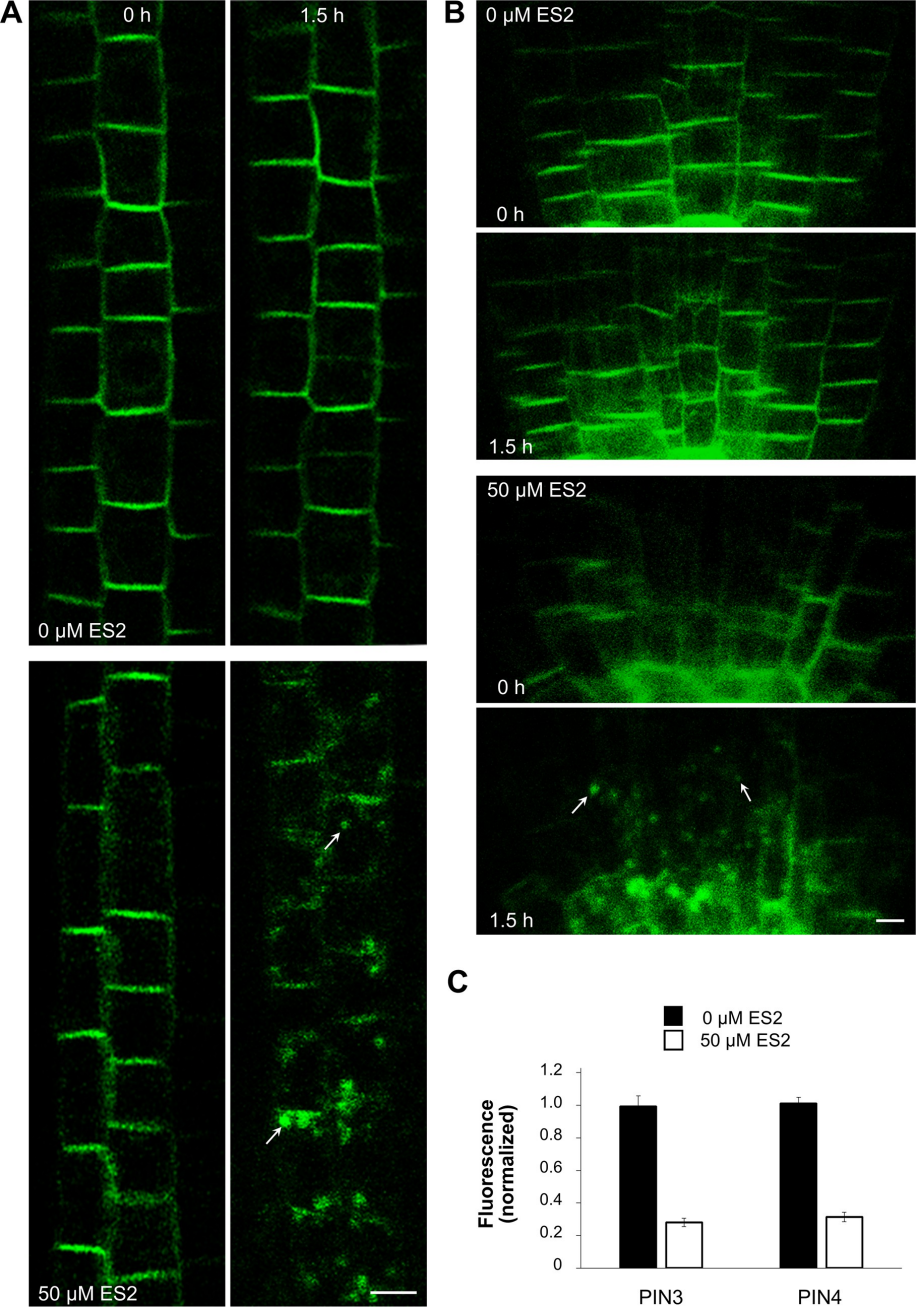
PIN3 and PIN4 are sensitive to ES2 treatment. When seedlings were treated with 50 μM ES2 for 1.5 hours, ES2As were visible in the endodermal cells of PIN3-Dendra2 seedling roots (arrows in A) and root meristem cells adjacent to the root cap of PIN4-Dendra2 plants (arrows in B). Roots were imaged and then reimaged after 1.5 hours of treatment with 50 μM ES2. The chart in C depicts relative fluorescence intensities for the PM. The mean of green signal intensities after 1.5 hours of treatment with ES2 was normalized to the mean of green signal intensity of the PM of the same root before ES2 application. T-test shows statistically highly significant differences in the PM signal intensity between ES2 treated and untreated samples (in both cases p ≤ 0.001, 14 roots were analyzed for PIN3, 12 roots for PIN4) Bars = 5 μm.

**Fig 7:**
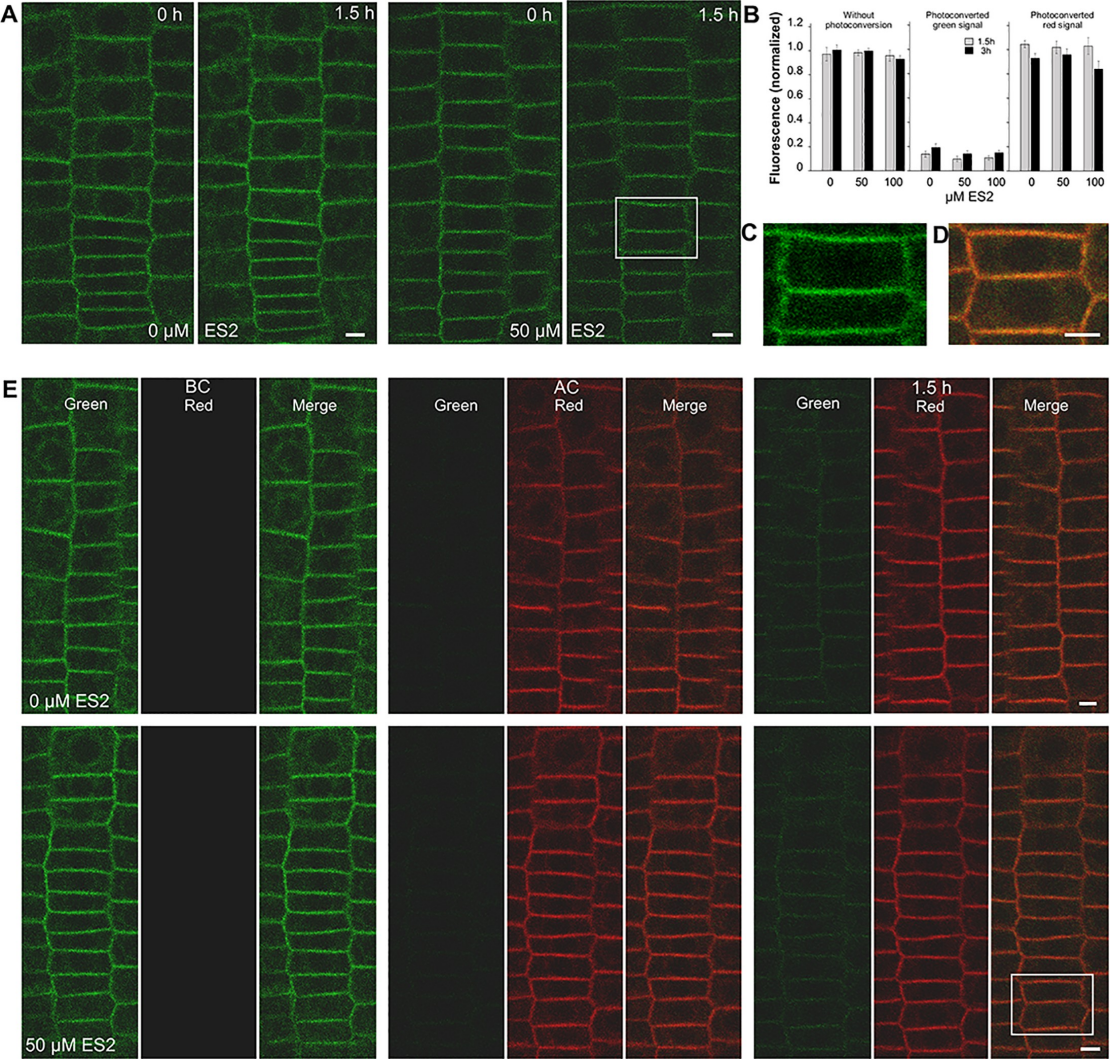
Endosidin2 has no apparent effect on SYT1 dynamics. In the experiment without photoconversion, roots were placed on the medium with different ES2 concentrations, immediately imaged and then reimaged after 1,5 and 3 hours. In the experiment with photoconversion, seedlings were placed on the medium with different concentrations of ES2, then roots were immediately imaged, photoconverted, imaged again, and reimaged after 1.5 and 3 hours. A figure shows a representative image taken during experiments without photoconversion, B shows an image captured during the experiment with photoconversion, C and D show magnified images of the rectangle areas in A and E, respectively. Note that the SYT1-Dendra2 fusion protein does not accumulate in vacuoles or aggregate in the cytoplasm. The B chart shows relative PM fluorescence intensities after 1.5 and 3 hours of treatment with 50 or 100 μM ES2. In the experiment without photoconversion, the means of the PM's signal intensities in roots were normalized to the mean of signal intensities of the PM of the same root before ES2 application. In the photoconversion experiment, the means of PM green signal intensities in the roots after the photoconversion were normalized to the mean of green signal intensities of the PM of the same root before photoconversion (BC in the figure). The means of red signal intensities were normalized to the mean red fluorescence intensity measured immediately after photoconversion (AC in the figure). The two-way ANOVA test showed statistically significant differences among time points (p ≤ 0.05, n = 15) but no significant effect of ES2 treatment on green and red signal intensities in the photoconverting experiment. No effects were found in the experiment without photoconversion. Bars = 5 μm.

The pioneering study by Zhang et al. [6] demonstrated that PIN2, PIN1, and BR1 proteins appear in ES2As, but other PM proteins such as PGP4 and PIP2a were not present in these aggregates. We checked other PINs, namely PIN3 and PIN4 and SYT1, a member of the Arabidopsis SYNAPTOTAGMIN family [16]. PIN3-Dendra2 was expressed in the endodermis, and it was visible in the ES2As developed in these cells (Fig 6A). PIN4-Dendra2 was dominantly expressed in the root cap, and to a lesser extent, in the central cylinder and meristematic cells close to the root cap. Under standard imaging conditions and without ES2 treatment, bodies appeared sporadically in the root cap, but we noticed no increase in the frequency of these structures after ES2 application (not shown). However, we observed ES2As labeled with PIN4-Dendra2 in meristematic cells (Fig 6B). The accumulation of PINs in cytoplasmic conglomerates was accompanied by the significantly decreased signal intensity in the PM of corresponding cells (Fig 6A–6C). SYT1-Dendra2 was profusely expressed in meristematic cells, and the protein was present in the PM and, less abundantly, also in the endomembrane system (Fig 7A, see also our previous study [16]). In the experiment without photoconversion, we could not distinguish any ES2As or vacuoles labeled by SYT1-Dendra2 (Fig 7A–7C), and record decreased signal intensity in the PM (Fig 7B). In the experiment with photoconversion, we failed to find a significant inhibitory effect of ES2 on green signal recovery in the PM or the accelerating effect of ES2 on red signal disappearing from the PM (Fig 7B, 7D and 7E). Moreover, we noticed neither red/green aggregates in the cytoplasm nor vacuole labeling (Fig 7D and 7E).

## Discussion

The application of small biologically active molecule is a useful and frequently employed approach in membrane trafficking studies [4,5]. However, it is not easy to identify target proteins, and off-target effects are often observed. ES2 [6] and its more active derivate ES2-14 isolated recently [7] belong to chemical substances that influence membrane protein dynamics and cause their entrapment in cytoplasm aggregates. Herein, we used the photoconvertible fluorescence protein technology and established that the trafficking routes of both newly synthesized and endocytic PIN2 populations are affected by ES2. In fact, the compact and strongly fluorescent bodies, previously described after ES2 application [6] and visible easily at low magnification in our routine time-lapse experiments, always contained both the PIN2 populations and the FM4-64 endocytic membrane tracer. However, the signals did not completely overlap in these structures, as demonstrated by Fig 4. The presence of the endocytic PIN2 population in ES2A is unexpected.

Interestingly, we previously demonstrated that both PIN2 pools are also trapped in BFACs [11,12]. In that case, a surprising result was the presence of the newly synthesized PIN2 form because BFA was previously supposed to block exclusively the re-secretion of endocytic PM proteins back to the PM [17–20]. However, comparison of data presented in this study and previous descriptions of BFACs demonstrates that the BFACs and ES2As are morphologically different in the epidermal Arabidopsis root cells. BFACs are large vesicle clusters surrounded by morphologically unaffected Golgi stacks [20,21]. On the contrary, after ES2 treatment, we observed the development of relatively small bodies that accumulate PIN protein populations. On the ultrastructural level, we detected distinct, tightly packed conglomerates composed of enlarged and abnormally cup-shaped Golgi units and their derivatives. Importantly we have found that pretreatment of cells with ES2 results in the absence of PIN2 in BFCs. This finding and previous observation by Huang and Zhang [22] suggest that ES2-sensitive processes are essential for gathering proteins in BFACs. We propose that ES2As are also composed of TGN/early endosome (TGN/EE) elements feeding into BFACs.

Several studies with pharmacological and genetic approaches describe anomalies in Golgi structure comparable to those revealed after ES2 application. Golgi stacks composed of extended curved, circular, or cup-shaped cisternae, were detected, e.g., in tobacco cells treated with 1-butanol [23], in *nevershed* [24] and double *mtv1/new* Arabidopsis mutants [25], in Arabidopsis with defect V-ATPase enzyme complex [26,27] or in Arabidopsis lines with two or three mutated *SYP4* genes [28]. In these studies, it was concluded that Golgi/TGN defects were caused by the disruption of transport vesicle biogenesis and trafficking that is in good agreement with our findings.

Importantly our comprehensive experiments with ES2 at high resolution and detector sensitivity have shown the presence of small, numerous, and weakly fluorescent clusters alongside large ES2As. In the photoconversion experiment, we have noticed red and green patches that rarely overlap. A similar pattern of signals was reported in cells co-expressing TGN/EE and Golgi reporters [29,30]. We suppose that the small, weakly green fluorescent clumps are the same aberrantly enlarged Golgi stacks seen with TEM. The Golgi function is affected by ES2, and therefore Golgi protein release is sufficiently hampered for the fluorescent tag to mature and to be visible in the Golgi. The red patches labeled by the PM-derived PIN2 population can be aberrant TGN/EE depicted by TEM as clumps of enlarged vesicles visible near the Golgi stacks. TGN/EEs are known to be involved in endocytic traffic (31), Large ES2As are apparently formed by aggregation of the green and red clumps. We suppose that ES2As represent conglomerates of aberrant Golgi/TGN units detected with TEM (Fig 5D and 5E).

It is noteworthy that PIN2 has become visible in vacuoles after ES2 treatment, where, as is known, it undergoes degradation [31]. Increased abundance of PIN2 in the vacuolar system was described after moving seedlings to the dark [31,32], and application of auxin [33] or gibberellic acid [34]. In the photoconversion experiment, we observed even a newly synthesized green PIN-Dendra2 in addition to its red variety in these structures. The vacuolar presence of an internalized red population derived from the PM is logical because the PIN2 turnover is very rapid, with a half-life of 3.5–4 hours [10], and cellular protein breakdown should be massive. However, why the newly synthesized green PIN2 population is redirected to the vacuole system after the ES2 treatment is unknown. Generally, mechanisms regulating membrane protein sorting from the TGN/EE compartments where endocytic and secretory traffic routes merge [35] to proper destinations are poorly understood in plants. Apparently, in plant cells, the TGN/EE is a complex heterogeneous system that includes subdomains or completely different TGN/EE subpopulations, which may respond differently to specific drug treatment [36]. Functional diversification of TGN/EE subpopulations may explain why PIN2 forms and FM64 fill different punctate and why they do not co-localized entirely in large ES2As in merge images.

The open question is whether there are other targets of ES2 besides Exo70. If ES2 acts in the cells exclusively through EXO70 mediating the assembly of the exocyst complex at the PM [37], the accumulation of post-Golgi secretory vesicles and their potential aggregation at the periphery of the cells would certainly be expected. Moreover, the vesicles containing PIN2 should logically accumulate in the polar position. Indeed, in exo70 mutant yeast, numerous 80–100 nm large post-Golgi vesicles were polarized to emerging buds and exocyst subunits, including EXO70, were mostly detected in the polar position [38]. We did not confirm polar localization of spots or large aggregates labeled by PIN2-Dendra2, and these were evenly scattered in the cytoplasm. Moreover, TEM showed no preferential accumulation of vesicles at the cell periphery. In contrast, a few vesicles of comparable size to that seen in the yeast exo70 mutants were always surrounding the Golgi. This finding suggests that ES2 inhibits not only late, as it has been suggested [6,7], but also earlier stages of Golgi and/or TGN derived vesicle traffic routes. It remains unclear if this occurrence is due to malfunctioning exocysts. Excysts' functional diversification and possible coordination of vesicle tethering to upstream vesicle-budding from the TGN and recycling endosomes remain subject to speculation [8,9]. However, it cannot be ruled out that EXO70 proteins have specific functions which are not limited by their role as exocyst complex subunits. Evidence supporting this idea includes localization studies, which reveal that a single representative of the EXO70 protein family has distinct cellular localization patterns [39].

Interestingly regard to our finding the Biogrid database (https://thebiogrid.org) indicates that in animals EXO70 physically interacts with proteins such as golgins, COG6, BIG2 or SEC 22, which are considered to be essential for many events connected with Golgi, including Golgi structure maintenance, and post Golgi vesicle trafficking [40–45]. The disruption of these associations could explain some anomalies of Golgi/TGN caused by ES2 treatment. Regarding protein sorting to vacuoles, there are confirmed interactions between EXO70 and proteins involved in the biogenesis of lysosome-related organelles, endosome sorting and trafficking, protein targeting for lysosome degradation or autophagosome initiation and maturation. However, functional analysis of most of these interactions has not been performed, and the EXO 70 interaction network in plants remains entirely unknown.

The challenging topic for further study is why ES2 selectively affects the trafficking of PM proteins. All PINs studied up to know [6, and this study] are affected with ES2. However, other proteins containing several transmembrane domains like PINs such as PLASMA MEMBRANE INTRINSIC PROTEIN 2a or P-GLYCOPROTEIN 4 are not influenced by ES2 (6). Here we show that AtSYT1, the integral PM protein with one N-terminally localized transmembrane domain [16], is also not affected by ES2A. AtSYT1 was suggested to move to the PM by convention secretion; however, it is unclear if this protein passes through TGN [46]. The present study also suggests that AtSYTs populations are not redirected to vacuoles for degradation. Perhaps this protein undergoes the breakdown differently. E.g., animal SYNAPTOTAGMIN IV was shown to be degraded by the proteasomal system [47].

In conclusion, it is plausible that ES2 effects on cells are complex. Here we indicate that ES2 affect both endocytic and exocytic pathway of PIN2. The open question is whether ES2 affects all processes by the same mechanism and through a dysfunctional exocyst due to its EXO70 subunits' malfunction. Most likely, this compound also acts beyond interference with EXO70. Moreover, Golgi/TGN appears to be the only cell structure affected by ES2 in epidermal root cells, but ES2’s precise biochemical mechanisms that cause morphological aberrations in these structures still require elucidation. Likely, ES2 inhibits vesicle development at the trans-Golgi and TGN. Golgi cisternae expand considerably as the membrane-flow from the endoplasmic reticulum to Golgi continues.

## Material and methods

### Cloning strategy and creation of transgenic lines

The PIN2-Dendra2 line was described in our previous study [10], and the translational fusion constructs of AtPIN3 (AT1G70940), AtPIN4 (AT2G01420) auxin transporters with DNA encoding Dendra2 photoconvertible protein [48] were prepared analogously. Briefly, the DNA sequences of PINs, including upstream and downstream regulatory sequences, were amplified by PCR from the genomic DNA of Arabidopsis thaliana (Col-0) and the Dendra2 sequence from Gateway®Dendra2-At-N entry clone (Evrogen, Moscow, Russia). We cloned fragments sequentially into the pAMPAT-MSC vector (GenBank: AY436765.1) so that Dendra2 was inserted in large cytoplasmic loops of PINs. The DNA sequence of SYNAPTOTAGMIN 1 (SYT1, At2g20990), including the upstream regulatory sequence, was also amplified by PCR from the genomic DNA of Arabidopsis (Col-0) and cloned into the pAMPAT-MSC vector. The DNA sequence encoding Dendra2 was attached to the 3ʼ end of SYT1 DNA. Sequences of primers are accessible in S1 Table. Arabidopsis (Col-0) plants were transformed by the floral dip method [49] using Agrobacterium tumefaciens GV3101 (pMP90RK) [50]. Finally, homozygous plants were selected with 7.5 mg/l phosphinothricin (PPT, Duchefa, Haarlem, The Netherlands).

### Plant material, cultivation, media, chemicals, and treatment

Seeds were surface sterilized with 1.5% (v/v) sodium hypochlorite and germinated on the ½ MSMO medium (Sigma, #M6899) supplemented with 1% (w/v) sucrose and solidified with 7 g/L Difco agar. Media pH was adjusted to 5.7, and Petri dishes were kept vertically in a growth chamber at 21°C and 100 μmol m^-2^ s^-1^ continuous light for four days. We initially transferred seedlings to fresh medium for 12 hours and then placed four to five seedlings on a microscopic slide covered with 1.5 solidified medium containing different compounds for the trials. Experiments were in triplicate. FM4-64 (Thermo Fisher Scientific) was used at two μM; cycloheximide, BFA, and ES2 (all obtained from Sigma-Aldrich) were at 50 μM dose unless otherwise stated. ES2 and BFA were dissolved in DMSO, and the other reagents were dissolved in water. The final concentration of DMSO in the medium was 0.1% (v/v). We re-checked the medium’s pH after drug supplementation. Slides with seedlings were placed on the solidified medium in Petri dishes and kept in the growth chamber under light for the entire experiment.

### Photoconversion and microscopy

We routinely removed coverslips after each microscopy imaging because the recovery of the PIN2 PM pool is suppressed in covered samples [10]. Experiments were performed mostly in both photoconversion and non-photoconversion mode. Photoconversion of Dendra2 was achieved by illuminating roots with a 100 W mercury lamp for 15 seconds with BP 400–410 nm filter and a 40X UPLSAPO Super Apochromat (0.90) objective of an Olympus FV1000 confocal laser scanning microscope. For routine time-lapse tests, two-channel image stacks were acquired sequentially in the multi-track mode under the same microscope at 20X Uplan FI objective (0.50 NA). The green signal was excited with a 488 nm line of an argon laser, and the signal was collected with a 505–525 nm band-pass filter. The red signal was excited with a 543 nm line of a HeNe laser, and fluorescence was collected with a 560–620 nm band-pass filter. We separated fluorescence emissions by a 488/543/633 nm dichroic mirror and adjusted the laser power and detector settings in such a way that the PM green signal fluorescence intensity before the conversion was proportional to the red signal intensity after conversion when both were analyzed by ImageJ package (National Institutes of Health, Bethesda, USA). We used the same LSM detector and laser line power gain and offset parameters throughout the time-lapse experiment. For detailed co-localization analysis, we imaged the root tips by high-resolution Leica TCS SP8 STED 3X confocal laser scanning microscope after excitation from the pulsed white laser WLL2. Here, green Dendra2 was excited with 491nm, and the red Dendra2 and FM4-64 were excited by 523 nm laser line and HC PL APO CS2 63X oil objective (1.40 NA Oil). Signals emitted by the green Dendra2, red Dendra 2 and FM 4–64 were determined by hybrid detectors in the ranges of 500–520 nm, 560–600 nm, and 680–750 nm, respectively. The confocal aperture was set at one Airy unit, thus providing x-y resolution of ~175 μm and z-resolution of ~ 500 μm. Z stacks of images with plane separation of 0.15 μm were collected by SuperZ galvanometric stage. In co-localization experiments, the red PIN2-Dendra2 population was excited at 553 nm. Other parameters were as described above.

### TEM

For ultrastructural studies, 4-day-old seedlings were treated with 50 μM ES2 or 50 μM BFA for half, one and two hours, and fixed for four hours with 3% (w/v) glutaraldehyde and 1% paraformaldehyde in 50 mM MTSB buffer [51]. After washing in MTSB, the buffer samples were postfixed with 1% (v/v) osmium tetroxide for two hours in the same buffer. Samples were then dehydrated in ethanol and embedded in Spurr's resin [52]. Ultra-thin sections were cut by LKB 8800 Ultratome III, stained with uranyl acetate and lead citrate [53] and estimated by Tesla BS 500 electron microscope.

### Experimental design, data evaluation, processing, and presentation

We measured the mean fluorescence intensities by ImageJ software. We employed the straight line for the transversal epidermal PM of the roots and the freehand selection tool for BFACs and ES2As. We investigated 15 to 20 membranes at each time point in every 12–15 experimental roots. Graph results are presented as relative values unless otherwise stated, and the methods used for normalizing fluorescence signals are described in the figure legends. The data points in plots prepared by Microsoft Excel (Microsoft, Redmond, USA) present the means of 12–15 plant roots’ normalized values. The bars correspond to the standard errors of means, and the time points in the graphs indicate when images were captured during the experiment. The figure images were processed by ImageJ, Adobe Photoshop CS2 (Adobe Systems, Mountain View, USA), and Microsoft Publisher software (Microsoft, Redmond, USA). The Z-stack images captured by Leica TCS SP8 STED 3X confocal laser scanning microscope were deconvoluted using the classic maximum likelihood estimation in Huygens Professional software (Scientific Volume Imaging B.V., Netherlands). Co-localization analysis was calculated by the Pearson correlation coefficient in ImageJ software. We analyzed approximately 30 optical slices per treatment in ROI covering entire similar-sized cytoplasm areas or in 70X70 (2.91 X 2.91μm) pixel-sized ROI for co-localization of large individual ES2As.

## Supporting information

S1 Fig ES2 induces aggregation of PIN2-Dendra2 in the cytoplasm and appearance of the fusion protein in the vacuole. In A, roots were treated with 50 μM ES2, in B and C co-treated with 50 μM ES2 and 2 μM FM4-6. Fluorescence signal in ES2As (arrowheads) is stronger than in vacuole-like structures (asterisks) in root cells treated with ES2 for 1.5 hours (A). The small spots labeled with FM4-64 (red signal) and PIN2-Dendra2 (green signal) developed within 30 minutes of ES2 and FM4-64 co-treatment rarely overlap (B). In cells co-treated for three hours, the vacuole (asterisk) accumulating PIN2-Dendra2 fusion protein is surrounded by the tonoplast labeled by FM4-64 (arrows in C). Note that the large ES2As (arrowheads) are seen in both channels. Bars = 5 μm.
(PDF)

S2 Fig Gallery of optical Z stack showing the distribution of PIN2 populations in the cells after ES2 treatment. Roots were illuminated to photoconvert fusion protein and treated with 50 μM ES2. Both secretory (green fluorescence) and endocytic (red fluorescence) PIN2-Dendra2 populations appear in the large intra-cytoplasmic ES2As (arrowheads) and vacuoles (two-headed arrows in optical section No. 3) 1.5 hours after photoconversion and ES2 application. Note that some ES2As occur in vacuoles or their vicinity (arrows in optical sections Nos. 1, 4, and 5). Numerous small spots seen in green and red channels are also present in the cells. In optical sections, Nos. 8–12, the double arrowheads indicate a couple of these partly overlapping bodies. The outlined area in the optical section No. 11 is presented at higher magnification and resolution in Fig 2B. Bars = 5 μm.
(PDF)

S3 Fig FM4-64 membrane tracer and PIN2 are captured together in large aggregates but not in small bodies. Roots were co-treated with 50 μM ES2 and 2 μM FM4-6 in the experiment without photoconversion. Large ES2As are co-labeled with the FM4-6 4 dye and PIN2-Dendra2 (arrows), but small spots are typically seen either in the green or red channel (arrowheads in optical section No. 5). The gallery shows the optical Z stack collected after 1.5 hours of ES2 treatment. Bars = 5 μm.
(PDF)

S4 Fig PIN2 and FM4-64 do not co-localize entirely in cells treated with cycloheximide. After 30 minutes of co-treatment with 50 μM ES2 and 2 μM FM4-64 in the presence of 50 μM cycloheximide, the endocytosed PIN2-Dendra2 and FM4-64 do not usually co-localize (A); However, both PIN2 and FM4-64 occur in large ES2As visible in the cells after 1.5 hours of co-treatment (B). The gallery at the bottom of figure B shows a magnified area of ES2A marked by the arrow in an upper merged image. The numbers represent the order of optical slices in the Z-stack image. Note, the only partial overlapping signal is seen in the ES2A (whitish regions). Bars = 2 μm.
(PDF)

S5 Fig ES2 effects are partly reversible. Washing ES2 out of the roots causes partial recovery of the PIN2 level in the PM (A to D). Roots were placed on the medium with 50 μM ES2 and imaged (-2 h). After 2 hours of treatment, seedlings were rinsed shortly with the liquid medium without ES2, placed on the ES2-free medium, immediately pictured (0 h), and re-imaged at different time points. Image A shows a representative root, images B and C are at higher magnification and show the cell delineated in panel A. The Chart in D illustrates changes in the PM fluorescence intensities during the experiment. T-test shows a statistically significant difference in the PM fluorescence intensity between time points 0 and 1 (p ≤ 0.001, 12 roots were analyzed). E and F demonstrate the effect of ES2 on root elongation. In this experiment, seedlings were germinated on the standard medium for four days, and then placed on medium with 20 or 50 μM ES2. After two hours, seedlings were placed on the ES2-free medium; alternatively, they were kept permanently on the medium with ES2. The chart in F depicts increasing root lengths after 24 and 48 hours. Representative seedlings treated continuously with ES2 for 48 hours are shown in E. The one-way Anova test showed the statistically significant effect of ES2 in both experiments after 24 and 48 hours of ES2 treatment (p ≤ 0.001, n = 20, the experiment was repeated with similar results) The Bars = 5 μm.
(PDF)

S6 Fig ES2As and BFACs have different developmental parameters. Originally relatively small BFACs (A shows BFACs induced by 20 min treatment with 50 μM BFA) enlarge quickly, and only one or two large BFACs are present in the cells after 1.5 hours treatment (C, D). ES2As remain relatively small (B displays the cells after 1.5 hours of treatment with 50 μM ES2) regardless of the concentration or duration of ES2 treatment (C). The ultrastructure of the root epidermal cell after 1.5-hours of BFA treatment is shown in E (arrows point to BFACs). In F, seedlings were treated with ES2 for 1.5 hours and then co-treated with ES2 and BFA for 1 hour. In G seedlings were treated with BFA for 1.5 hours and then co-treated with ES2 and BFA for 1 hour. Note that in F, the large fluorescent compartments are absent. Bars = 5 μm.
(PDF)

S1 Table Primers used to prepare DNA constructs. (PDF)
