# Supplementary material for: Endosidin 2 accelerates PIN2 endocytosis and disturbs intracellular trafficking of PIN2, PIN3, and PIN4 but not of SYT1
Source: PLoS One. 2020 Aug 13;15(8):e0237448. doi: 10.1371/journal.pone.0237448 (PMC7425933; doi:10.1371/journal.pone.0237448)
Supplement: S1 Fig — In A, roots were treated with 50 μM ES2, in B and C co-treated with 50 μM ES2 and 2 μM FM4-6. Fluorescence signal in ES2As (arrowheads) is stronger than in vacuole-like structures (asterisks) in root cells treated with ES2 for 1.5 hours (A). The small spots labeled with FM4-64 (red signal) and PIN2-Dendra2 (green signal) developed within 30 minutes of ES2 and FM4-64 co-treatment rarely overlap (B). In cells co-treated for three hours, the vacuole (asterisk) accumulating PIN2-Dendra2 fusion protein is surrounded by the tonoplast labeled by FM4-64 (arrows in C). Note that the large ES2As (arrowheads) are seen in both channels. Bars = 5 μm. (PDF) [file pone.0237448.s001.pdf]

Supporting information

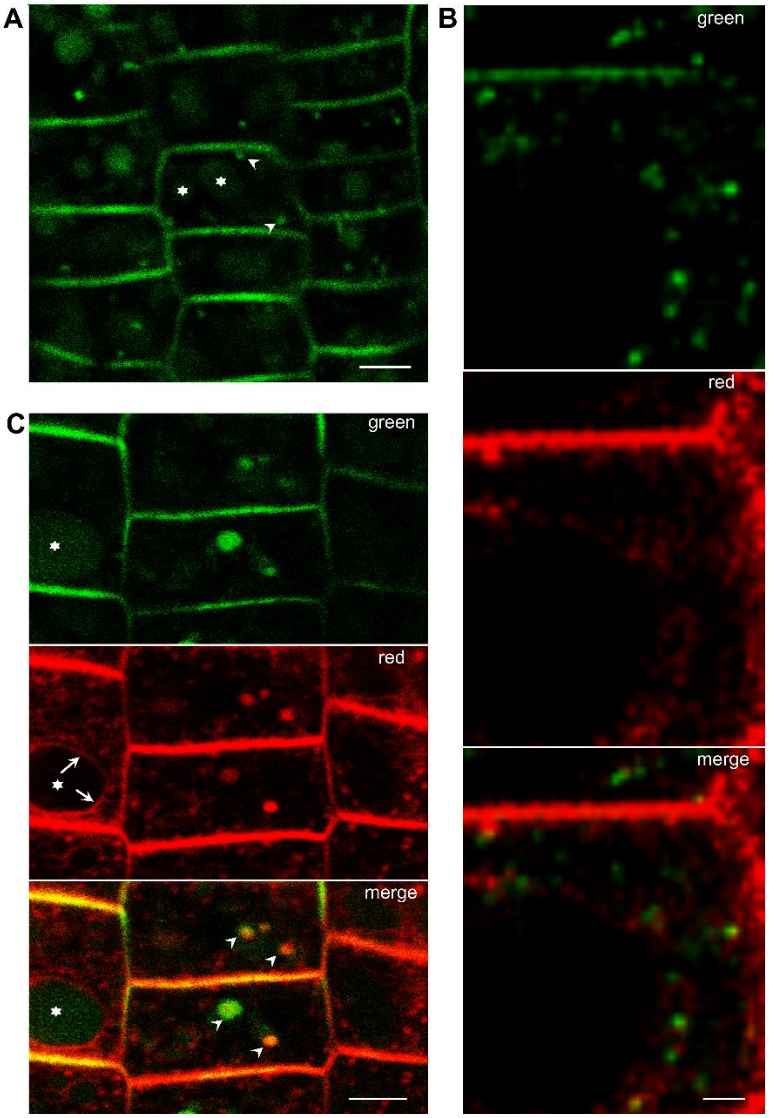

S1 Fig. ES2 induces aggregation of PIN2-Dendra2 in the cytoplasm and appearance of the fusion protein in the vacuole.

In A, roots were treated with 50 μM ES2, in B and C co-treated with 50 μM ES2 and 2 μM FM4-6. Fluorescence signal in ES2As (arrowheads) is stronger than in vacuole-like structures (asterisks) in root cells treated with ES2 for 1.5 hours (A). The small spots labeled with FM4-64 (red signal) and PIN2-Dendra2 (green signal) developed within 30 minutes of ES2 and FM4-64 co-treatment rarely overlap (B). In cells co-treated for three hours, the vacuole (asterisk) accumulating PIN2-Dendra2 fusion protein is surrounded by the tonoplast labeled by FM4-64 (arrows in C). Note that the large ES2As (arrowheads) are seen in both channels. Bars = 5 μm.
