# Supplementary material for: Endosidin 2 accelerates PIN2 endocytosis and disturbs intracellular trafficking of PIN2, PIN3, and PIN4 but not of SYT1
Source: PLoS One. 2020 Aug 13;15(8):e0237448. doi: 10.1371/journal.pone.0237448 (PMC7425933; doi:10.1371/journal.pone.0237448)
Supplement: S2 Fig — Roots were illuminated to photoconvert fusion protein and treated with 50 μM ES2. Both secretory (green fluorescence) and endocytic (red fluorescence) PIN2-Dendra2 populations appear in the large intra-cytoplasmic ES2As (arrowheads) and vacuoles (two-headed arrows in optical section No. 3) 1.5 hours after photoconversion and ES2 application. Note that some ES2As occur in vacuoles or their vicinity (arrows in optical sections Nos. 1, 4, and 5). Numerous small spots seen in green and red channels are also present in the cells. In optical sections, Nos. 8–12, the double arrowheads indicate a couple of these partly overlapping bodies. The outlined area in the optical section No. 11 is presented at higher magnification and resolution in Fig 2B. Bars = 5 μm. (PDF) [file pone.0237448.s002.pdf]

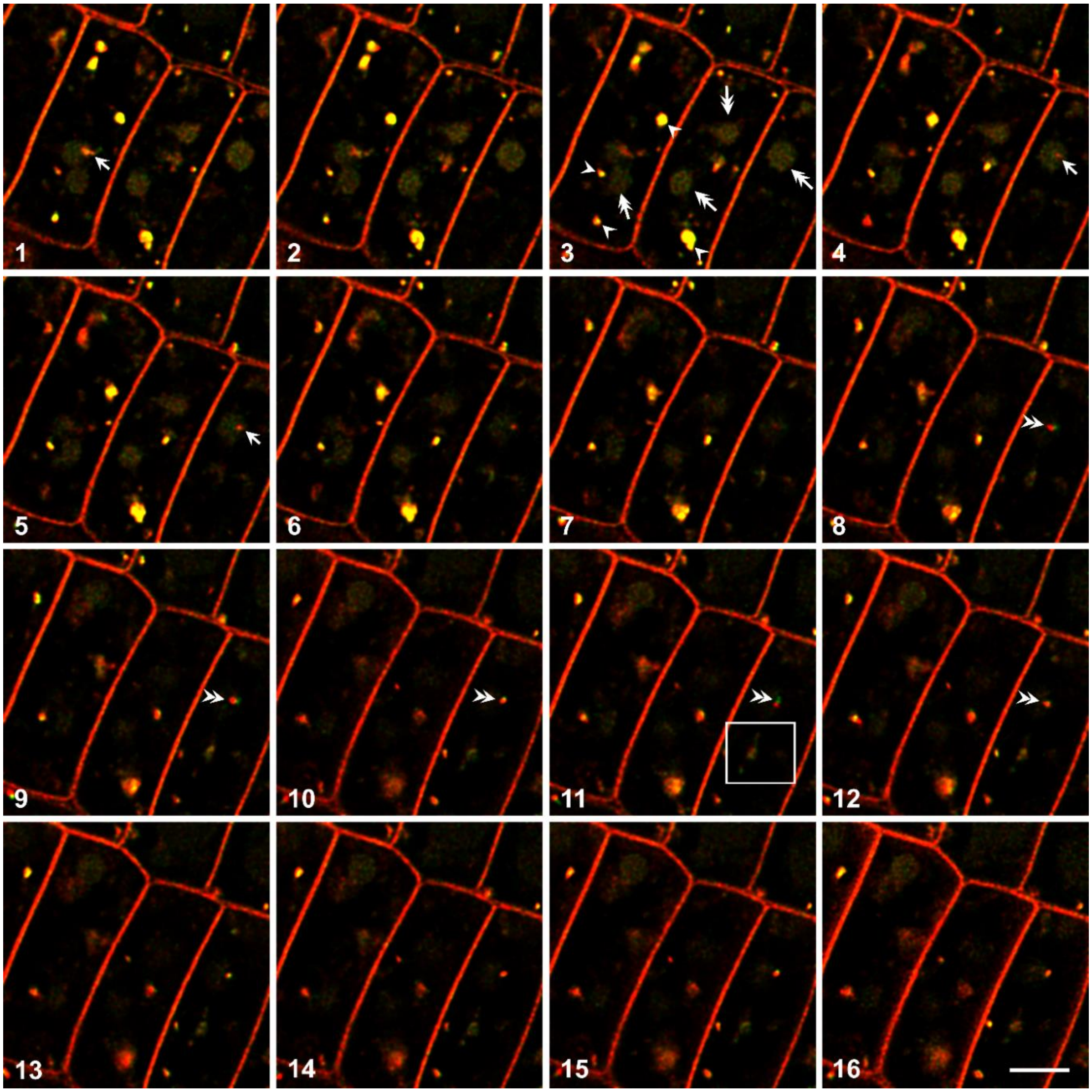

S2 Fig. Gallery of optical Z stack showing the distribution of PIN2 populations in the cells after ES2 treatment.

Roots were illuminated to photoconvert fusion protein and treated with 50 μM ES2. Both secretory (green fluorescence) and endocytic (red fluorescence) PIN2-Dendra2 populations appear in the large intra-cytoplasmic ES2As (arrowheads) and vacuoles (two-headed arrows in optical section No. 3) 1.5 hours after photoconversion and ES2 application. Note that some ES2As occur in vacuoles or their vicinity (arrows in optical sections Nos. 1, 4, and 5). Numerous small spots seen in green and red channels are also present in the cells. In optical sections, Nos. 8-12, the double arrowheads indicate a couple of these partly overlapping bodies. The outlined area in the optical section No. 11 is presented at higher magnification and resolution in Fig. 2 B. Bars = 5 μm.
