# Supplementary material for: Endosidin 2 accelerates PIN2 endocytosis and disturbs intracellular trafficking of PIN2, PIN3, and PIN4 but not of SYT1
Source: PLoS One. 2020 Aug 13;15(8):e0237448. doi: 10.1371/journal.pone.0237448 (PMC7425933; doi:10.1371/journal.pone.0237448)
Supplement: S3 Fig — Roots were co-treated with 50 μM ES2 and 2 μM FM4-6 in the experiment without photoconversion. Large ES2As are co-labeled with the FM4-6 4 dye and PIN2-Dendra2 (arrows), but small spots are typically seen either in the green or red channel (arrowheads in optical section No. 5). The gallery shows the optical Z stack collected after 1.5 hours of ES2 treatment. Bars = 5 μm. (PDF) [file pone.0237448.s003.pdf]

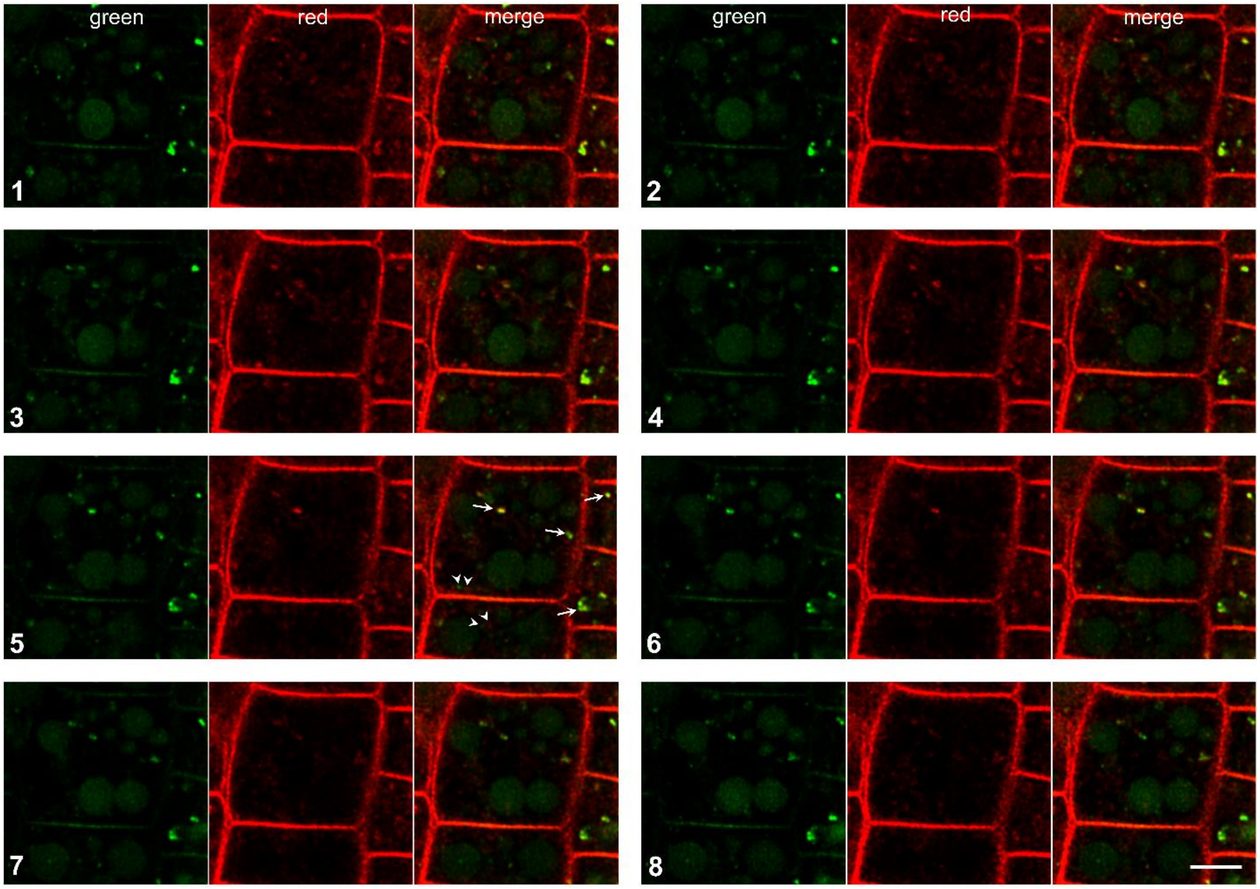

S3 Fig. FM4-64 membrane tracer and PIN2 are captured together in large aggregates but not in small bodies.

Roots were co-treated with 50 μM ES2 and 2 μM FM4-6 in the experiment without photoconversion. Large ES2As are co-labeled with the FM4-6 4 dye and PIN2-Dendra2 (arrows), but small spots are typically seen either in the green or red channel (arrowheads in optical section No. 5). The gallery shows the optical Z stack collected after 1.5 hours of ES2 treatment. Bars = 5 μm.
