# Supplementary material for: Endosidin 2 accelerates PIN2 endocytosis and disturbs intracellular trafficking of PIN2, PIN3, and PIN4 but not of SYT1
Source: PLoS One. 2020 Aug 13;15(8):e0237448. doi: 10.1371/journal.pone.0237448 (PMC7425933; doi:10.1371/journal.pone.0237448)
Supplement: S4 Fig — After 30 minutes of co-treatment with 50 μM ES2 and 2 μM FM4-64 in the presence of 50 μM cycloheximide, the endocytosed PIN2-Dendra2 and FM4-64 do not usually co-localize (A); However, both PIN2 and FM4-64 occur in large ES2As visible in the cells after 1.5 hours of co-treatment (B). The gallery at the bottom of figure B shows a magnified area of ES2A marked by the arrow in an upper merged image. The numbers represent the order of optical slices in the Z-stack image. Note, the only partial overlapping signal is seen in the ES2A (whitish regions). Bars = 2 μm. (PDF) [file pone.0237448.s004.pdf]

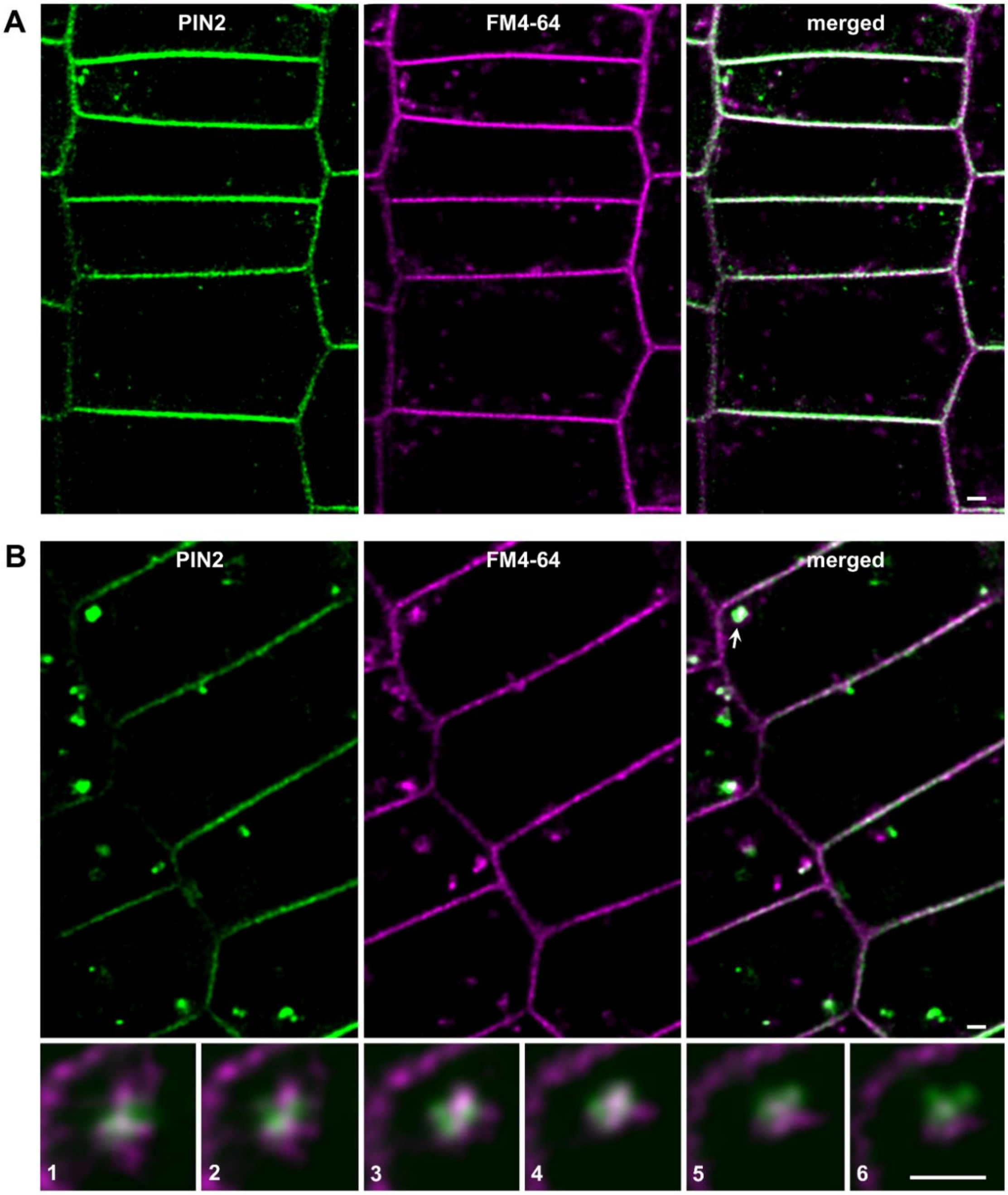

S4 Fig. PIN2 and FM4-64 do not co-localize entirely in cells treated with cycloheximide.

After 30 minutes of co-treatment with 50 μM ES2 and 2 μM FM4-64 in the presence of 50 μM cycloheximide, the endocytosed PIN2-Dendra2 and FM4-64 do not usually co-localize (A); However, both PIN2 and FM4-64 occur in large ES2As visible in the cells after 1.5 hours of co-treatment (B). The gallery at the bottom of figure B shows a magnified area of ES2A marked by the arrow in an upper merged image. The numbers represent the order of optical slices in the Z-stack image. Note, the only partial overlapping signal is seen in the ES2A (whitish regions). Bars = 2 μm.
