# Supplementary material for: Endosidin 2 accelerates PIN2 endocytosis and disturbs intracellular trafficking of PIN2, PIN3, and PIN4 but not of SYT1
Source: PLoS One. 2020 Aug 13;15(8):e0237448. doi: 10.1371/journal.pone.0237448 (PMC7425933; doi:10.1371/journal.pone.0237448)
Supplement: S5 Fig — Washing ES2 out of the roots causes partial recovery of the PIN2 level in the PM (A to D). Roots were placed on the medium with 50 μM ES2 and imaged (-2 h). After 2 hours of treatment, seedlings were rinsed shortly with the liquid medium without ES2, placed on the ES2-free medium, immediately pictured (0 h), and re-imaged at different time points. Image A shows a representative root, images B and C are at higher magnification and show the cell delineated in panel A. The Chart in D illustrates changes in the PM fluorescence intensities during the experiment. T-test shows a statistically significant difference in the PM fluorescence intensity between time points 0 and 1 (p ≤ 0.001, 12 roots were analyzed). E and F demonstrate the effect of ES2 on root elongation. In this experiment, seedlings were germinated on the standard medium for four days, and then placed on medium with 20 or 50 μM ES2. After two hours, seedlings were placed on the ES2-free medium; alternatively, they were kept permanently on the medium with ES2. The chart in F depicts increasing root lengths after 24 and 48 hours. Representative seedlings treated continuously with ES2 for 48 hours are shown in E. The one-way Anova test showed the statistically significant effect of ES2 in both experiments after 24 and 48 hours of ES2 treatment (p ≤ 0.001, n = 20, the experiment was repeated with similar results) The Bars = 5 μm. (PDF) [file pone.0237448.s005.pdf]

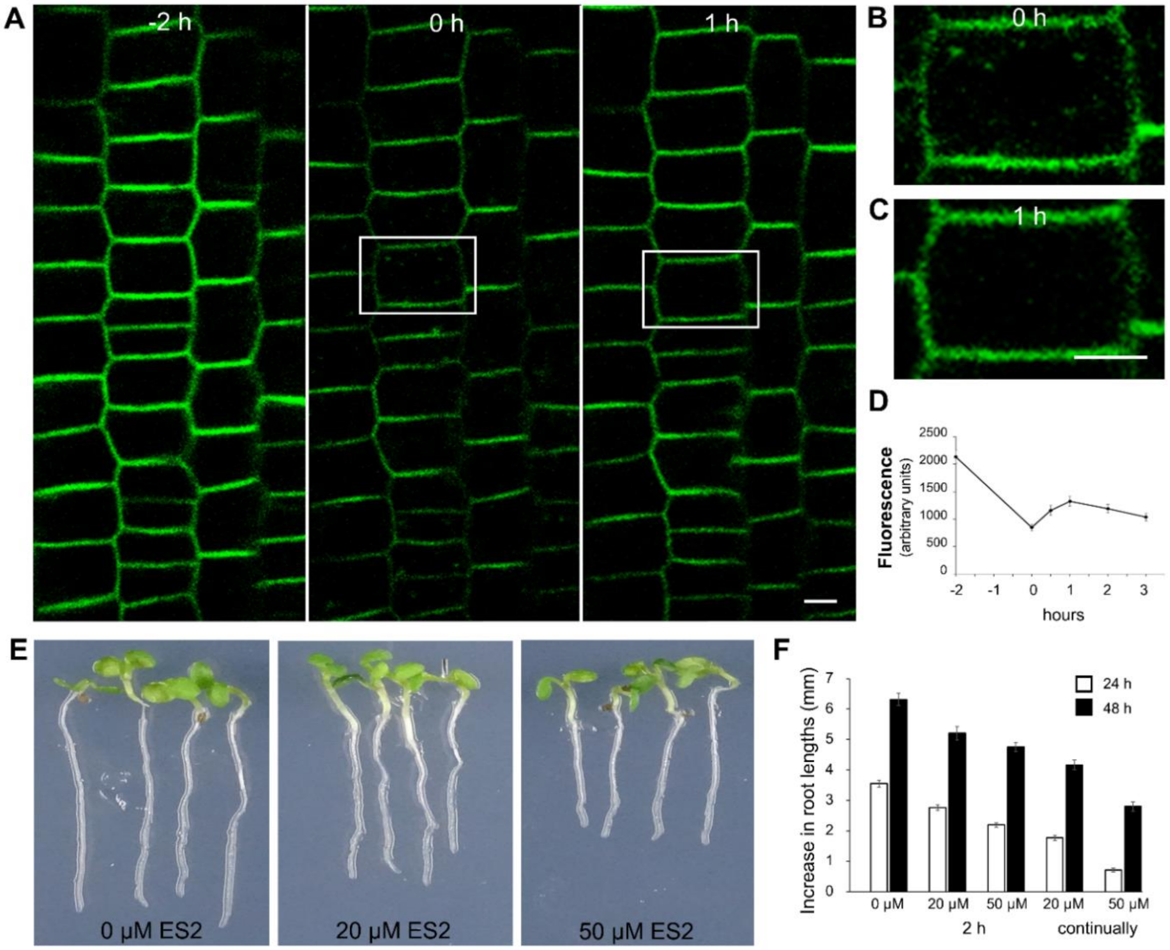

S5 Fig. ES2 effects are partly reversible.

Washing ES2 out of the roots causes partial recovery of the PIN2 level in the PM (A to D). Roots were placed on the medium with 50 µM ES2 and imaged (-2 h). After 2 hours of treatment, seedlings were rinsed shortly with the liquid medium without ES2, placed on the ES2-free medium, immediately pictured (0 h), and re-imaged at different time points. Image A shows a representative root, images B and C are at higher magnification and show the cell delineated in panel A. The Chart in D illustrates changes in the PM fluorescence intensities during the experiment. T-test shows a statistically significant difference in the PM fluorescence intensity between time points 0 and 1 ($p \le 0.001$, 12 roots were analyzed). E and F demonstrate the effect of ES2 on root elongation. In this experiment, seedlings were germinated on the standard medium for four days, and then placed on medium with 20 or 50 µM ES2. After two hours, seedlings were placed on the ES2-free medium; alternatively, they were kept permanently on the medium with ES2. The chart in F depicts increasing root lengths after 24 and 48 hours. Representative seedlings treated continuously with ES2 for 48 hours are shown in E. The one-way Anova test showed the statistically significant effect of ES2 in both experiments after 24 and 48 hours of ES2 treatment ($p \le 0.001$, $n = 20$, the experiment was repeated with similar results) The Bars = 5 µm.
