# Supplementary material for: Endosidin 2 accelerates PIN2 endocytosis and disturbs intracellular trafficking of PIN2, PIN3, and PIN4 but not of SYT1
Source: PLoS One. 2020 Aug 13;15(8):e0237448. doi: 10.1371/journal.pone.0237448 (PMC7425933; doi:10.1371/journal.pone.0237448)
Supplement: S6 Fig — Originally relatively small BFACs (A shows BFACs induced by 20 min treatment with 50 μM BFA) enlarge quickly, and only one or two large BFACs are present in the cells after 1.5 hours treatment (C, D). ES2As remain relatively small (B displays the cells after 1.5 hours of treatment with 50 μM ES2) regardless of the concentration or duration of ES2 treatment (C). The ultrastructure of the root epidermal cell after 1.5-hours of BFA treatment is shown in E (arrows point to BFACs). In F, seedlings were treated with ES2 for 1.5 hours and then co-treated with ES2 and BFA for 1 hour. In G seedlings were treated with BFA for 1.5 hours and then co-treated with ES2 and BFA for 1 hour. Note that in F, the large fluorescent compartments are absent. Bars = 5 μm. (PDF) [file pone.0237448.s006.pdf]

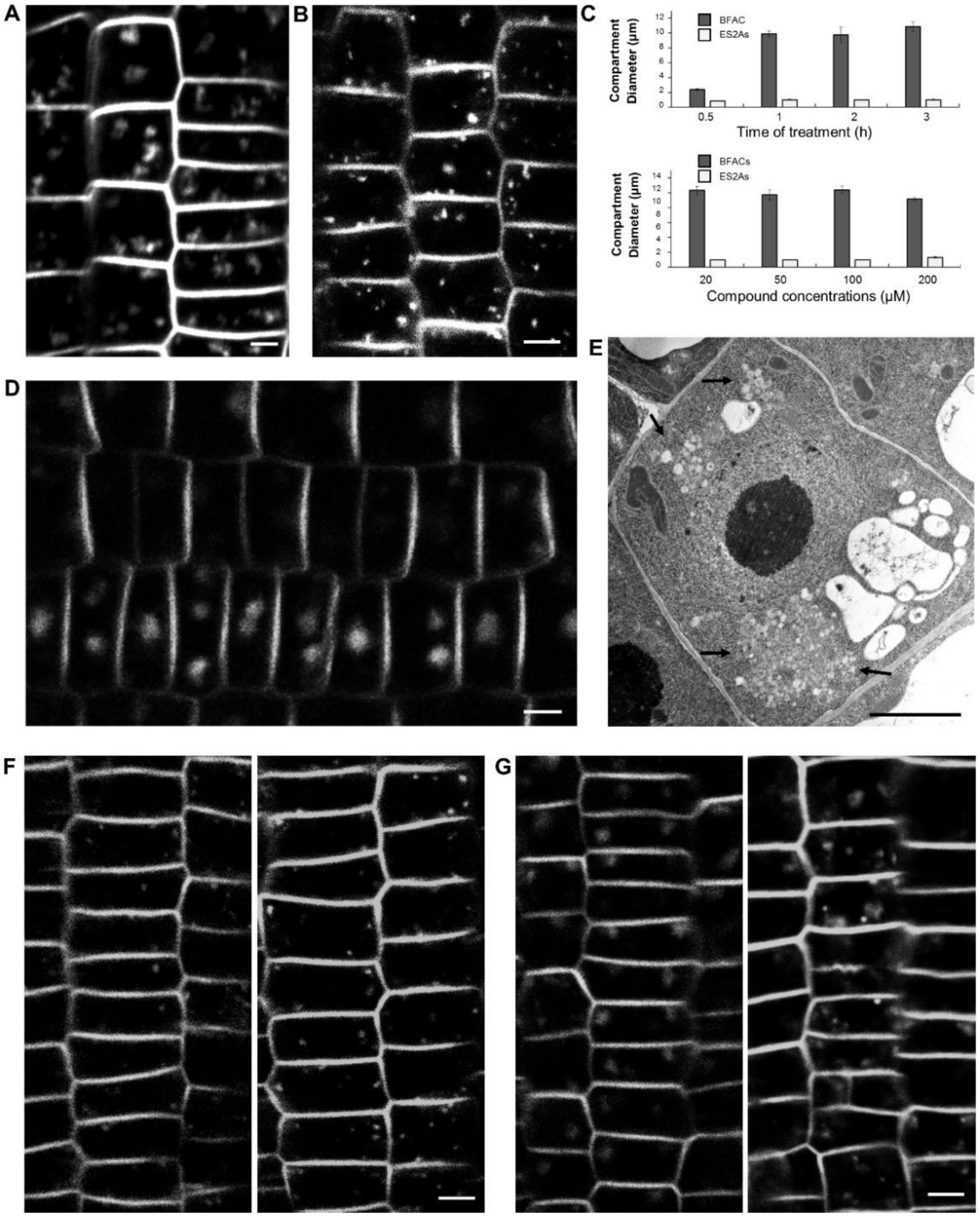


**S6 Fig. ES2As and BFACs have different developmental parameters.**

Originally relatively small BFACs (A shows BFACs induced by 20 min treatment with 50 µM BFA) enlarge quickly, and only one or two large BFACs are present in the cells after 1.5 hours treatment (C, D). ES2As remain relatively small (B displays the cells after 1.5 hours of treatment with 50 µM ES2) regardless of the concentration or duration of ES2 treatment (C). The ultrastructure of the root epidermal cell after 1.5-hours of BFA treatment is shown in E (arrows point to BFACs). In F, seedlings were treated with ES2 for 1.5 hours and then co-treated with ES2 and BFA for 1 hour. In G seedlings were treated with BFA for 1.5 hours and then co-treated with ES2 and BFA for 1 hour. Note that in F, the large fluorescent compartments are absent. Bars = 5 µm
