# Supplementary material for: Endosidin 2 accelerates PIN2 endocytosis and disturbs intracellular trafficking of PIN2, PIN3, and PIN4 but not of SYT1
Source: PLoS One. 2020 Aug 13;15(8):e0237448. doi: 10.1371/journal.pone.0237448 (PMC7425933; doi:10.1371/journal.pone.0237448)
Supplement: S1 Table — (PDF) [file pone.0237448.s007.pdf]

pAtPIN2::AtPIN2-5′ end::Dendra2::AtPIN2-3′ end::pAAtPIN2 cassette (abbreviated as PIN2-Dendra2)

PIN2-F1	AAAAAGGCGCGCCAAAGATGAAGAAACCGTATAAAGG
PIN2-R1	AACCTGAATTCATAGCATTCTTCGCGTTGGCTTC
PIN2-F2	CACGACTGCAGCTATGACCAGAGGTTCTTCCACCGA
PIN2-R2	ATATTGCGGCCGCTTTAATAACATGCTTCACGACCATCG
DEND2-F1	CGGTGAATTCCATGAACACTCCTGGAATCAATCTC
DEND2-R1	ACACTTGAGTCTGCAGACCAAACCTGT

pAtPIN3::AtPIN3-5′ end::Dendra2::AtPIN3-3′ end::pAAtPIN3 cassette (abbreviated as PIN3-Dendra2)

PIN3-F1	TCTGGGCGCGCCATTTACTCATGTGAAACTTTGGCT
PIN3-R1	TGTCTTGGAATTCAGCGCCGCCGTGGAATTTGGA
PIN3-F2	TCCAAATTCCACTGCAGCGCTACAATCCAAGACA
PIN3-R2	CATTTGCGGCCGCAGAAAAGAATCAGTAAAGCCACCA
DEND2-F1	CGGTGAATTCCATGAACACTCCTGGAATCAATCTC
DEND2-R1	ACACTTGAGTCTGCAGACCAAACCTGT

pAtPIN4::AtPIN4-5′ end::Dendra2::AtPIN4-3′ end::pAAtPIN4 cassette (abbreviated as PIN4-Dendra2)

PIN4-F1	TAGGGGCGCGCCTCGTCTGCTTCTCTGATATTTCC
PIN4-R1	TTTAGAATTCCCTGCTGTAGCTTTCTCTATCTCTC
PIN4-F2	GAGAAAGCTACTGCAGGGCTGAATAAAATGG
PIN4-R2	TCCTGCGGCCGCATAATGGTTCATGAATGGCTCTCTC
DEND2-F1	CGGTGAATTCCATGAACACTCCTGGAATCAATCTC
DEND2-R1	ACACTTGAGTCTGCAGACCAAACCTGT

pAtSYT1::AtSYT1::Dendra2 (abbreviated as SYT1-Dendra2)

SYT1-F	GTTGGCGCGCCACCGAAAACCGTTCCTGCCTTTTCT
SYT1-R	GCAATGAATTCAGAGGCAGTTCGCCACTCGAGCTC
DEND2-F2	GATATGAATTCGGCGCCGGCATGAACACTCCTGGAATCAATCTC
DEND2-R2	CAAGGATCCTCACCAAACCTGTGATGGGAGAG

S1 Table. Primers used to prepare DNA constructs.
